# Cheese-whey permeate improves the fitness of *Escherichia coli* cells during recombinant protein production

**DOI:** 10.1186/s13068-023-02281-8

**Published:** 2023-02-23

**Authors:** Marcella de Divitiis, Diletta Ami, Alex Pessina, Alessandro Palmioli, Barbara Sciandrone, Cristina Airoldi, Maria Elena Regonesi, Luca Brambilla, Marina Lotti, Antonino Natalello, Stefania Brocca, Marco Mangiagalli

**Affiliations:** Department of Biotechnology and Biosciences, University of Milano-Bicocca, Piazza Della Scienza 2, 20126 Milan, Italy

**Keywords:** Waste valorization, Dairy industry, Micro-FTIR, *Escherichia coli* BL21 (DE3), pET expression system, Recombinant proteins

## Abstract

**Background:**

*Escherichia coli* cells are the most frequently used hosts in recombinant protein production processes and mainly require molecules such as IPTG or pure lactose as inducers of heterologous expression. A possible way to reduce the production costs is to replace traditional inducers with waste materials such as cheese whey permeate (CWP). CWP is a secondary by-product generated from the production of the valuable whey proteins, which are obtained from ultrafiltration of cheese whey, a main by-product of the dairy industry, which is rich in lactose.

**Results:**

The effects of CWP collected from an Italian plant were compared with those of traditional inducers on the production of two model proteins (i.e., green fluorescent protein and the toxic Q55 variant of ataxin-3), in *E. coli* BL21 (DE3) cells. It was found that the high lactose content of CWP (165 g/L) and the antioxidant properties of its micronutrients (vitamins, cofactors and osmolytes) sustain production yields similar to those obtained with traditional inducers, accompanied by the improvement of cell fitness.

**Conclusions:**

CWP has proven to be an effective and low-cost alternative inducer to produce recombinant proteins. Its use thus combines the advantage of exploiting a waste product with that of reducing the production costs of recombinant proteins.

**Supplementary Information:**

The online version contains supplementary material available at 10.1186/s13068-023-02281-8.

## Background

The zero-waste economy promoted by the EU requires the rational and sustainable use of natural resources and the reduction or valorization of waste often produced by traditional processes. Innovative technologies and processes have a key role in bioeconomy, to transform waste into secondary raw materials that will become the starting point for new production chains of new products, possibly with high added value [1–3].

Traditionally, the food sector, i.e., the activities related to food processing and consuming, produces a large amount of waste, whose composition and abundance makes it difficult to dispose and dump [4]. Agro-food waste produced by industries can accumulate at different stages of the process, including both harvest and processing [5]. Thus, although endowed with great nutritional potential, agro-food waste has often been considered an economic and environmental problem rather than a resource. Agro-food waste can be directly exploited as a component of functional food for human nutrition or animal feeding [6–8]. On the other hand, in a context of bioeconomy, agro-food waste can be valorised through the production of high-value products such as bioplastic, bioactive compounds and nutraceuticals [4, 9]. These processes require natural or metabolically modified microorganisms such as bacteria, yeasts and fungi, the so-called “microbial cell factories”, acting as “biorefineries” able to transform raw materials into high-value products [1, 4, 10].

Among agro-food waste, cheese whey (CW) represents one of the main polluting wastes due its abundance and high biochemical oxygen demand. CW is produced during the cheese making process and consists of the liquid phase generated after the separation of the curd. Generally, 1 kg of cheese is obtained from 10 kg of milk, generating 9 kg of CW, which is mainly composed of lactose (45–50 g/L), proteins (6–8 g/L), lipids (4–5 g/L), and minerals salts [11, 12]. In the last decades, CW received attention as a source of value-added products such as whey proteins, which are marketed as a dietary supplement [13]. Whey proteins are separated from CW by ultrafiltration, generating CW permeate (CWP), a very lactose-rich secondary by-product. CWP can be exploited as a carbon source to support the growth of microbial cell factories [12, 14, 15], and as an alternative inducer in the production of recombinant proteins [16–19]. However, the effects of CWP and its nutrients on *Escherichia coli* cells physiology and fitness are elusive and poorly investigated.

**Fig. 1:**
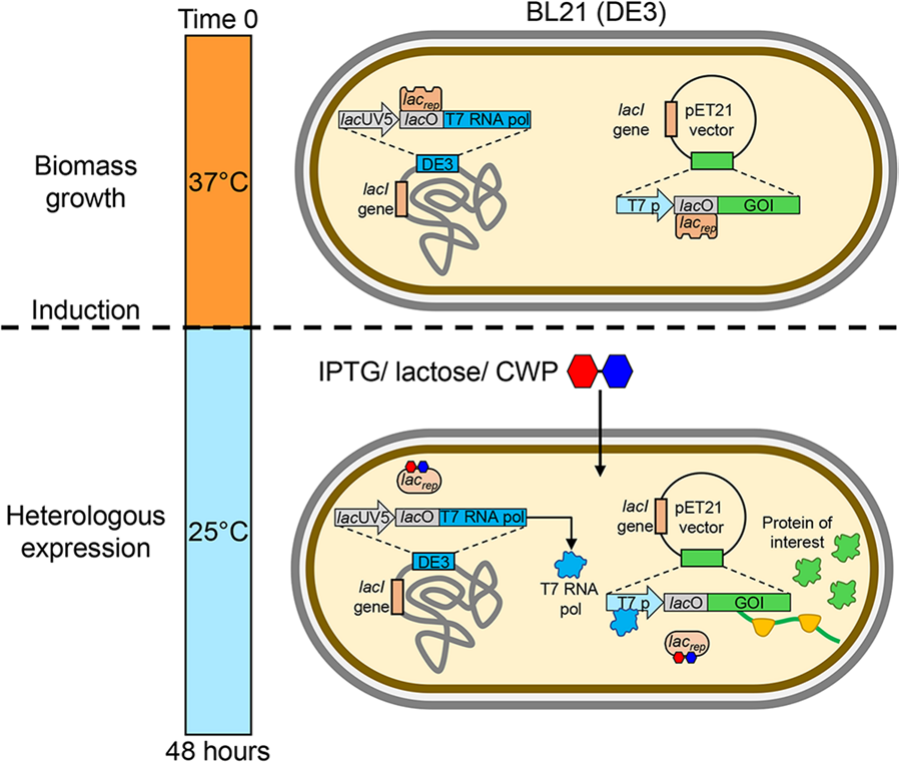
Experimental plan and BL21-pET expression systems. The production of GFP and ATX3-Q55 was carried out in two phases: (i) biomass growth and (ii) induction and recombinant production. During phase (i), the cells were grown at 37 °C without an inducer until reaching a cell density of OD_600_: 0.8–1. In these conditions, the lac repressor (*lac*_*rep*_) encoded by the *lacI* gene is bound to the lac operator (*lacO*) inhibiting the transcription of T7 RNA polymerase. The induction was carried out by adding IPTG (0.1 g/L), or lactose (10 g/L) or CWP (lactose: 10 g/L) and incubating the cells at 25 °C for 48 h. Adding the inducer to a growing culture of BL21 (DE3) cells induces the production of T7 RNA polymerase, which in turn transcribes the gene of interest (GOI) inserted into the plasmid

Recombinant proteins play an important role both in protein functional and structural studies and in applications such as the production of biopharmaceuticals and industrial enzymes [20–22]. Several different cell factories including bacteria, yeasts, insects and mammalian cells have been developed to support recombinant production of proteins [20–22]. *E. coli* cells are the most used microbial cell factories due to the ease in manipulation, the ability to grow on cheap substrates and to produce at high yields recombinant proteins [21, 22]. There is no doubt that among *E. coli* strains BL21 (DE3) and its derivatives (e.g., BL21 Rosetta, Origami and pLys) are the most used in the production of recombinant proteins. Developed by Studier and Moffat in 1986 [23], the BL21 (DE3) strain supports the use of expression vectors exploiting inducible promoters. More in detail, this strain harbors the gene coding for the T7 RNA polymerase under the control of the hybrid *lac*UV5 promoter. The genes of interest are usually cloned in suitable expression vectors, namely pET, under the control of the strong T7 phage promoter [24] and heterologous expression starts upon the addition of the inducer isopropyl β-d-thiogalactopyranoside (IPTG), which makes T7 RNA polymerase intracellularly available (Fig. 1). The BL21-pET system enables the production of recombinant proteins in two phases, one of biomass production, in the absence of inducers, and a subsequent induction phase; alternatively, it is possible to use so-called autoinduction media, which contain all the components of the culture medium, nutrients and inducers, from the start of cultivation [21, 21, 25]. Although BL21-pET systems are efficient and widely used to produce recombinant proteins at laboratory scale, the high costs of IPTG have limited their exploitation in large-scale industrial production [22]. To reduce production costs, pure lactose and CWP have already been used as alternative inducers, providing yields very similar to those obtained with IPTG [22].

Here, we describe the effects of CWP on cell physiology and recombinant production of two model proteins, namely green fluorescent protein (GFP) and the pathological ataxin-3 Q55 variant (ATX3-Q55). GFP was chosen because of its natural fluorescence, which makes it easily detectable, and because it is produced at high levels in *E. coli* cells as soluble protein [26, 27]. ATX3-Q55 consists of the globular Josephine N-terminal domain, followed by an unstructured C-terminal region containing 55 Gln residues. This protein was chosen for its toxicity and ability to cause stress in *E. coli* cells [28, 29]. CWP was obtained from an Italian company that collects and processes CW from the production of Grana Padano and Parmigiano Reggiano. Our data indicate that the yields of model proteins produced using CWP as an inducer are comparable to those observed with IPTG, and that CWP appears to alleviate the stresses caused by recombinant expression in host cells. Overall, CWP could be considered as an alternative inducer to IPTG, capable of improving process efficiency and reducing cellular stresses. Its use would make recombinant protein production processes more cost-efficient and eco-sustainable, thus realizing one of the key principles of the circular economy.

## Results

### CWP is rich in lactose and contains micronutrients

**Fig. 2:**
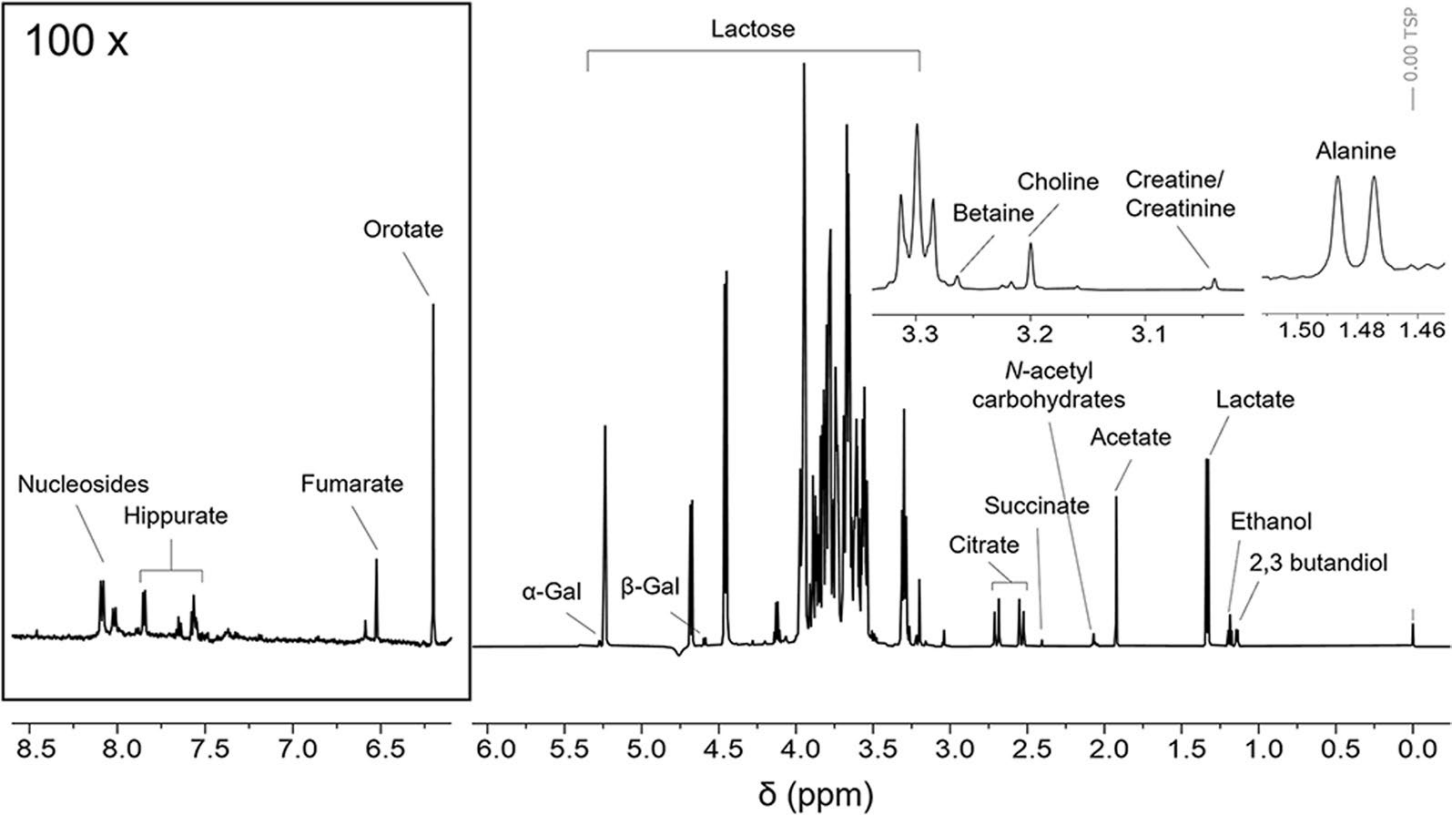
Characterization of CWP sample. ^1^H-NMR spectrum of CWP in d-PB 10 mM (TSP 1 mM, pH 7.4, 10% D_2_O) acquired at 25 °C and 600 MHz. Resonance assignments of the most important metabolites are reported

The chemical composition of CWP was investigated by an untargeted analytical methodology combining NMR spectroscopy and ultra-performance liquid chromatography separation coupled with high resolution mass spectrometry (UPLC–HRMS) analysis, recently adopted for the screening of complex mixtures such as foods and medicinal plants extracts [30–32]. NMR spectroscopy was exploited for qualitative and quantitative determination of major components in CWP sample, while the UPLC–HRMS data have been complementary used for qualitative identification of metabolites, including those ones that are below the detection limit of NMR analysis. The ^1^H-NMR spectrum of CWP sample is reported in Fig. 2; the detailed peak assignments and metabolite concentrations are reported in Table 1. The identification of metabolites was based on interpretation of ^1^H-NMR, ^1^H–^1^H-TOCSY and ^1^H–^13^C-HSQC spectra, then a specific library was built using the simple mixture analysis (SMA) tool implemented in Mestrenova 14.3. SMA allowed the semiautomatic identification and quantification of all metabolites contained in the mixture; the library developed with this approach was stored as .exp files [33] and will be free-available for future analysis of CWP samples. As expected, CWP is rich in lactose (165 g/L) and the presence of a small amount of galactose (3 g/L) was also determined. Moreover, several organic acids, such as lactate (5 g/L), citrate (4.8 g/L), acetate (1.2 g/L), succinate (0.09 g/L), hippurate (*N*-benzoylglycine, 0.06 g/L) and fumarate (0.02 g/L), and micronutrients such as choline (also known as vitamin B_4_, 0.4 g/L), creatine/creatinine (0.4 g/L), orotate (also known as vitamin B_13_, 0.26 g/L) and betaine (0.15 g/L) were found.

**Table 1:** Assignments of ^1^H resonances of identified metabolites and their concentration in CWP sample

Metabolite	^1^H chemical shift (ppm)	Concentration (g/L)
Lactose	5.24 (d, *J* = 3.8 Hz), 4.68 (d, *J* = 7.9 Hz), 4.46 (d, *J* = 7.8 Hz), 4.0–3.55 (m), 3.30 (m)	165.247
Lactate	1.33 (d, *J* = 7.0 Hz), 4.12 (q, *J* = 7.0 Hz)	5.094
Citrate	2.70 (d, *J* = 17 Hz), 2.54 (d, *J* = 17 Hz)	4.814
Galactose	5.27 (d, *J* = 3.9 Hz, H_1α_), 4.59 (d, *J* = 7.9 Hz, H_1β_)	3.055
Ethanol	1.19 (t, *J* = 7.1 Hz), 3.65 (m)	1.339
Acetate	1.91 (s)	1.196
Choline	3.19 (s)	0.406
Creatine/creatinine	3.04 (s)	0.399
2,3-butandiol	1.14 (d, *J* = 6.0 Hz), 3.73 (m)	0.354
Orotate	6.20 (s)	0.258
Betaine	3.26 (s)	0.150
Succinate	2.40 (s)	0.091
Hippurate	7.85 (d, *J* = 7.7 Hz), 7.65 (t, *J* = 7.7 Hz), 7.56 (m)	0.063
Alanine	1.47 (d, *J* = 7.3 Hz), 3.76 (m)	0.048
Fumarate	6.51 (s)	0.021

Peaks are reported as found in ^1^H-NMR and ^1^H,^1^H-TOCSY spectra of CWP sample (d-PB 10 mM, pH 7.4, 25 °C, TSP 1 mM)

s: singlet; d: doublet; t: triplet; m: multiplet

Moreover, UPLC–HRMS analysis allowed to the identification of 40 compounds, including primary and secondary metabolites, vitamins, such as riboflavin (also known as vitamin B_2_) and pantothenic acid (also known as vitamin B_5_), and coenzymes, such as flavin adenine dinucleotide. Detailed spectrometric data are reported in Table 2.

**Table 2:** UPLC/HR-MS data for the components identified in CWP sample

RT (min)	Average m/z	Metabolite name	Adduct type	Reference m/z	Error (ppm)	Formula	Ontology	INCHIKEY
1.07	89.0231	Lactate	[M−H]^−^	89.0229	3.0	C_3_H_6_O_3_	Alpha hydroxy acids and derivatives	JVTAAEKCZFNVCJ-REOHCLBHSA-N
1.12	117.0181	Succinate	[M−H]^−^	117.0182	− 0.7	C_4_H_6_O_4_	Dicarboxylic acids and derivatives	KDYFGRWQOYBRFD-UHFFFAOYSA-N
1.32	218.1384	Propionyl carnitine	[M+H]^+^	218.1387	− 1.5	C_10_H_19_NO_4_	Acyl carnitines	UFAHZIUFPNSHSL-UHFFFAOYNA-N
1.34	140.0338	6-Hydroxynicotinic acid	[M+H]^+^	140.0342	− 2.9	C_6_H_5_NO_3_	Pyridinecarboxylic acids	BLHCMGRVFXRYRN-UHFFFAOYSA-N
1.35	182.0452	4-Pyridoxic acid	[M−H]^−^	182.0451	0.8	C_8_H_9_NO_4_	Pyridinecarboxylic acids	HXACOUQIXZGNBF-UHFFFAOYSA-N
	184.0600		[M+H]^+^	184.0604	− 2.2			
1.53	283.0671	Xanthosin	[M−H]^−^	283.0680	− 3.3	C_10_H_12_N_4_O_6_	Purine nucleosides	UBORTCNDUKBEOP-UUOKFMHZSA-N
1.59	191.0186	Citric acid	[M−H]^−^	191.0191	− 2.5	C_6_H_8_O_7_	Tricarboxylic acids and derivatives	KRKNYBCHXYNGOX-UHFFFAOYSA-N
2.14	146.0597	2-Hydroxyquinoline	[M+H]^+^	146.0600	− 2.4	C_9_H_7_NO	Hydroquinolones	LISFMEBWQUVKPJ-UHFFFAOYSA-N
2.70	218.1025	Pantothenate (Vitamin B5)	[M−H]^−^	218.1034	− 4.0	C_9_H_17_NO_5_	Secondary alcohols	GHOKWGTUZJEAQD-ZETCQYMHSA-N
	220.1179		[M+H]^+^	220.1180	− 0.3			
2.85	232.1543	Butyryl carnitine	[M+H]^+^	232.1553	− 4.5	C_11_H_21_NO_4_	Acyl carnitines	LRCNOZRCYBNMEP-UHFFFAOYNA-N
3.26	162.0545	Indole-3-carboxylic acid	[M+H]^+^	162.0550	− 2.7	C_9_H_7_NO_2_	Indolecarboxylic acids and derivatives	KMAKOBLIOCQGJP-UHFFFAOYSA-N
	160.0392		[M−H]^−^	160.0394	− 1.2			
3.26	117.0549	2-Hydroxyvaleric acid	[M−H]^−^	117.0552	− 2.8	C_5_H_10_O_3_	Hydroxy fatty acids	JRHWHSJDIILJAT-UHFFFAOYSA-N
3.28	188.9857	2,5-Dihydroxybenzenesulfonate	[M−H]^−^	188.9863	− 3.4	C_6_H_6_O_5_S	Benzenesulfonic acids and derivatives	IKQCSJBQLWJEPU-UHFFFAOYSA-N
3.60	194.0446	4-Hydroxyhippuric acid	[M−H]^−^	194.0452	− 3.3	C_9_H_9_NO_4_	Hippuric acids	ZMHLUFWWWPBTIU-UHFFFAOYSA-N
3.63	203.0814	l-Tryptophan	[M−H]^−^	203.0819	− 2.4	C_11_H_12_N_2_O_2_	Indolyl carboxylic acids and derivatives	QIVBCDIJIAJPQS-VIFPVBQESA-N
3.73	181.0494	4-Hydroxyphenyllactic acid	[M−H]^−^	181.0498	− 2.0	C_9_H_10_O_4_	Phenylpropanoic acids	JVGVDSSUAVXRDY-UHFFFAOYSA-N
3.76	158.0812	*N*-Isovalerylglycine	[M−H]^−^	158.0810	1.5	C_7_H_13_NO_3_	*N*-acyl-alpha amino acids	ZRQXMKMBBMNNQC-UHFFFAOYSA-N
4.14	190.0488	Kynurenic acid	[M+H]^+^	190.0499	− 5.7	C_10_H_7_NO_3_	Quinoline carboxylic acids	HCZHHEIFKROPDY-UHFFFAOYSA-N
4.21	175.0599	2-Isopropylmalic acid	[M−H]^−^	175.0607	− 4.2	C_7_H_12_O_5_	Hydroxy fatty acids	BITYXLXUCSKTJS-UHFFFAOYSA-N
4.31	784.1497	FAD	[M−H]^−^	784.1500	− 0.4	C_27_H_33_N_9_O_15_P_2_	Flavin nucleotides	VWWQXMAJTJZDQX-UYBVJOGSSA-N
4.53	212.0014	3-Indoxylsulfate	[M−H]^−^	212.0018	− 1.7	C_8_H_7_NO_4_S	Arylsulfates	BXFFHSIDQOFMLE-UHFFFAOYSA-N
4.71	178.0503	Hippurate	[M−H]^−^	178.0510	− 3.9	C_9_H_9_NO_3_	Hippuric acids	QIAFMBKCNZACKA-UHFFFAOYSA-N
4.73	160.0393	2,8-Quinolinediol	[M−H]^−^	160.0395	− 1.2	C_9_H_7_NO_2_	Quinolones and derivatives	ZXZKYYHTWHJHFT-UHFFFAOYSA-N
	162.0545		[M+H]^+^	162.0550	− 2.9			
5.10	153.0183	2,3-Dihydroxybenzoate	[M−H]^−^	153.0182	1.0	C_7_H_6_O_4_	Salicylic acids	GLDQAMYCGOIJDV-UHFFFAOYSA-N
5.25	131.0704	2-Hydroxyisocaproic acid	[M−H]^−^	131.0709	− 3.7	C_6_H_12_O_3_	Hydroxy fatty acids	LVRFTAZAXQPQHI-UHFFFAOYSA-N
5.27	377.1453	Riboflavin	[M+H]^+^	377.1456	− 0.7	C_17_H_20_N_4_O_6_	Flavins	AUNGANRZJHBGPY-SCRDCRAPSA-N
	375.1303		[M−H]^−^	375.1310	− 1.9			
5.34	192.0658	Phenylacetylglycine	[M−H]^−^	192.0660	− 1.2	C_10_H_11_NO_3_	*N*-acyl-alpha amino acids	UTYVDVLMYQPLQB-UHFFFAOYSA-N
5.93	194.0449	alpha-Hydroxyhippuric acid	[M−H]^−^	194.0458	− 4.6	C_9_H_9_NO_4_	Hippuric acids	GCWCVCCEIQXUQU-UHFFFAOYSA-N
5.93	150.0550	*N*-Methylanthranilic acid	[M−H]^−^	150.0552	− 1.3	C_8_H_9_NO_2_	Aminobenzoic acids	WVMBPWMAQDVZCM-UHFFFAOYSA-N
6.06	147.0442	3-Isochromanone	[M−H]^−^	147.0443	− 0.9	C_9_H_8_O_2_	2-benzopyrans	ILHLUZUMRJQEAH-UHFFFAOYSA-N
6.06	165.0549	3-phenyllactic acid	[M−H]^−^	165.0557	− 4.7	C_9_H_10_O_3_	Phenylpropanoic acids	VOXXWSYKYCBWHO-UHFFFAOYSA-N
6.54	204.0662	Indolelactic acid	[M−H]^−^	204.0657	2.6	C_11_H_11_NO_3_	Indolyl carboxylic acids and derivatives	XGILAAMKEQUXLS-UHFFFAOYSA-N
7.06	204.0662	*N*-Cinnamoylglycine	[M−H]^−^	204.0658	2.1	C_11_H_11_NO_3_	*N*-acyl-alpha amino acids	YAADMLWHGMUGQL-VOTSOKGWSA-N
7.27	243.0869	7,8-dimethylalloxazine (lumichrome)	[M+H]^+^	243.0877	− 3.2	C_12_H_10_N_4_O_2_	Flavin derivative	ZJTJUVIJVLLGSP-UHFFFAOYSA-N
10.94	464.3009	Glycholate	[M−H]^−^	464.3010	− 0.1	C_26_H_43_NO_6_	Glycinated bile acids and derivatives	RFDAIACWWDREDC-RGWSMLPJSA-N

Finally, the CWP antioxidant capacity (AOC) was determined through the evaluation of the total reducing power (Folin-Ciocalteau assay) and the radical scavenging activity (ABTS and DPPH assays). Results are listed in Table 3. For comparison, an equivalent solution of lactose (170 g/L) was tested to investigate its contribution to AOC. Overall, CWP showed an average of 66 mg gallic acid equivalent per liter of sample as total reducing power, and 706 and 386 µmol of Trolox equivalent per liter of sample as radical scavenging activity, in ABTS and DPPH assays respectively. These results are significantly greater than those obtained with the equivalent lactose solution, highlighting the contribution of CWP components to AOC.

**Table 3:** Antioxidant activity of CWP

	Folin-Ciocalteau (GAE mg/L)	ABTS (TE µmol/L)	DPPH (TE µmol/L)
CWP	66.08 ± 1.81	706.04 ± 35.96	386.29 ± 27.58
Lactose (170 g/L)	4.74 ± 2.02	13.33 ± 1.65	7.25 ± 3.11

Results were expressed as gallic acid equivalent (GAE) and Trolox equivalent (TE) as mean (± SD) of triplicate measurements of two independent experiments (*n* = 6) and reported in comparison with an equivalent solution of lactose (170 g/L)

### CWP acts as an inducer of GFP expression and has beneficial effects on cell biomass production

The ability of CWP to replace traditional inducers used in the production of recombinant proteins, such as IPTG and lactose, was evaluated using two different model proteins, i.e., GFP and ATX3-Q55. Recombinant protein production was carried out in *E. coli* BL21(DE3) cells and the genes encoding the two model proteins were cloned in the pET21 plasmid. Expression conditions were first set up for GFP, which is easily produced as a soluble protein with high yields in *E. coli* BL21(DE3) cells. Moreover, its natural fluorescence allows us to easily monitor and quantitatively compare the overexpression of GFP following induction with lactose, CWP and IPTG as inducers. Cells were grown in Lennox medium modified with the addition of 5 g/L glycerol (LM-G), a sustainable carbon source suitable for bacterial culture media [34–36]. *E. coli* cells were cultured in LM-G medium at 37 °C until the exponential growth phase (OD_600_ ~ 1) was reached, when the inducer, namely IPTG (0.1 g/L), or lactose (10 g/L) or CWP (lactose: 10 g/L), was added, and the subsequent 48-h incubation at 25 °C began (Fig. 1).

**Fig. 3:**
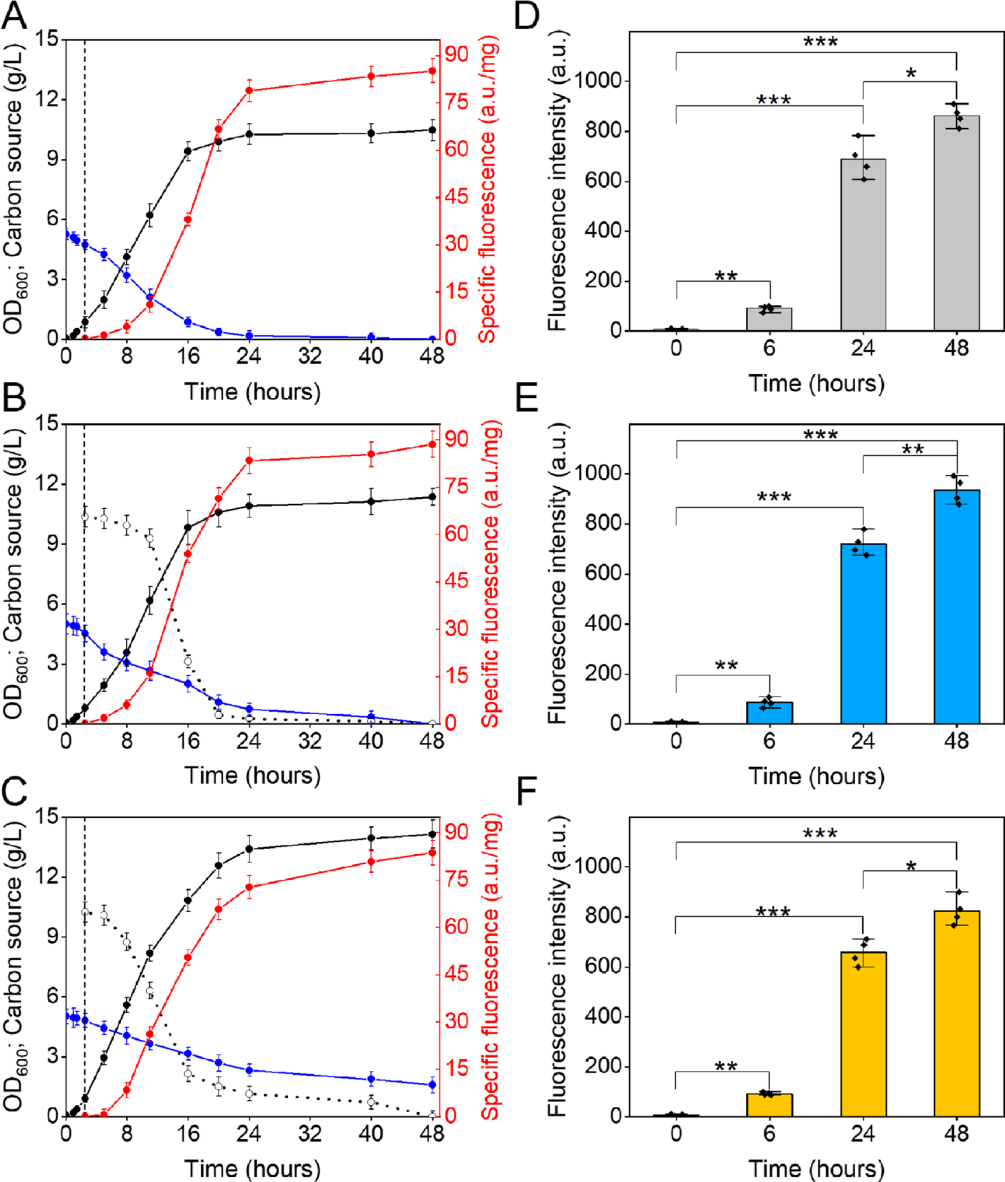
Effects of different inducers on GFP production. Growth curves of *E. coli* BL21 (DE3) cells overexpressing GFP upon induction with IPTG (**A**), lactose (**B**), and CWP (**C**), at 25 °C for 48 h. Cell density (black line, OD_600_), specific fluorescence (red line) and concentrations of glycerol (blue line) and lactose (dotted line) are plotted as a function of growth time. The vertical dashed line indicates the addition of the inducer, the error bars indicate the standard deviation of three independent measurements. **D**–**F** GFP production by a single cell was determined by flow cytometry from whole cells taken times after induction with IPTG (**D**), lactose (**E**), and CWP (**F**). The mean values of three (**A**–**C**) or four (**D**–**F**) independent measurements are represented with error bars indicating standard deviations. Statistical analyses were performed using unpaired two-tailed Student’s *t*-test, n.s.: not significant *p* > 0.05, *p < 0.05, **p < 0.01, ***p < 0.001

**Fig. 4:**
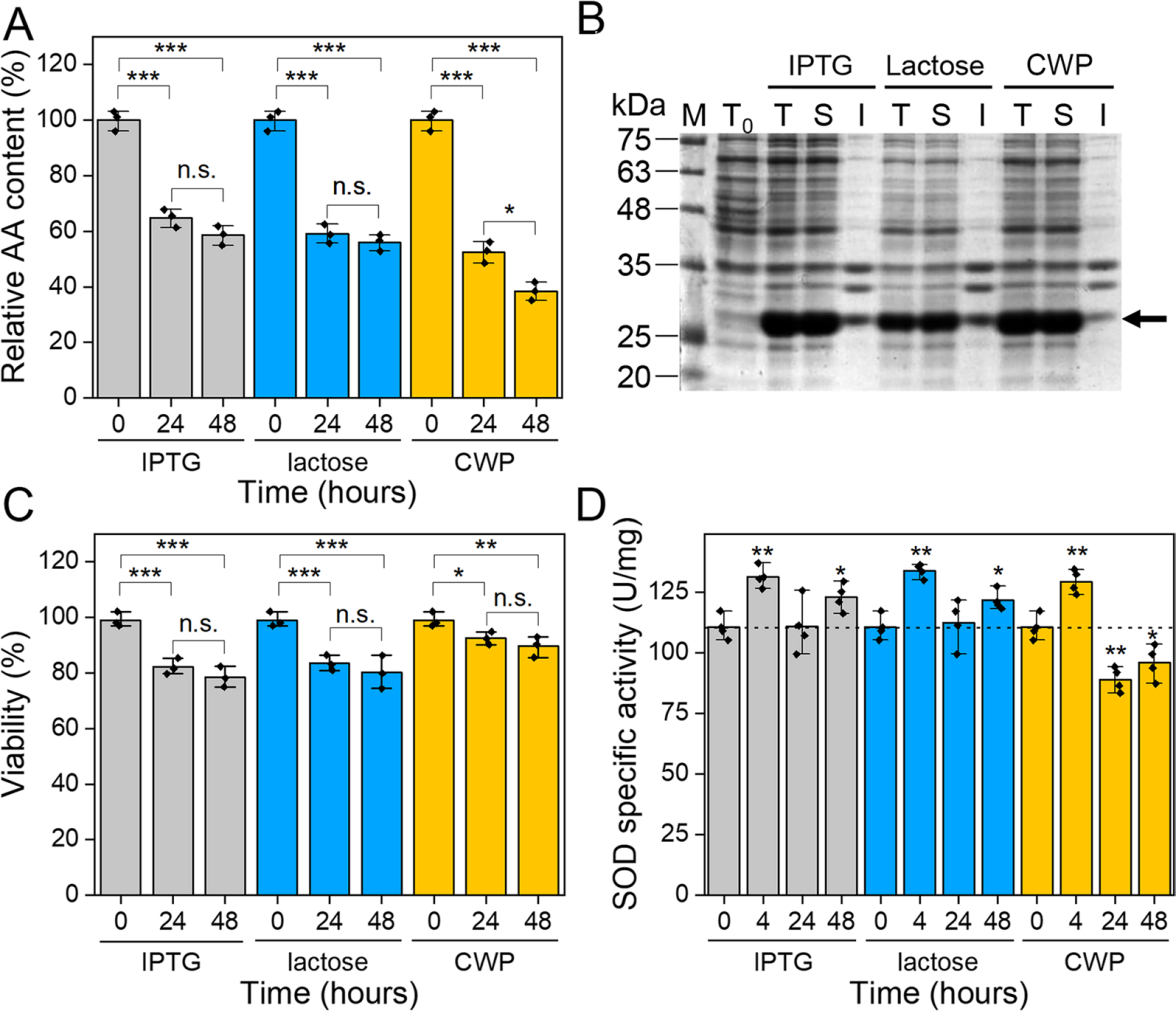
Effects of different inducers on cell physiology of GFP-producing cells. **A** Amino acid consumption determined by HPLC analysis of culture supernatants taken at the indicated post-induction times. **B** SDS-PAGE analysis of protein extracts from samples taken at the end of induction. M: molecular weight marker, *T*_0_: total protein fraction before the induction, *T*: total protein fraction, *S*: soluble protein fraction, *I*: insoluble protein fraction. **C** Cell viability determined by flow cytometry analysis after labeling dead cells with propidium iodine. **D** SOD specific activity measured by McCord and Fridovich assay on the soluble protein fraction extracted from samples taken at the indicated times. The mean values of three (**A**, **C**) or four (**D**) independent measurements are shown with error bars indicating standard deviations. Statistical analyses were performed using unpaired two-tailed Student’s *t*-test, n.s.: not significant *p* > 0.05, *p < 0.05, **p < 0.01, ***p < 0.001

The effects of the three different inducers were investigated by monitoring GFP production through its fluorescence signal, while cell biomass and nutrient consumption were quantified by OD_600_ and high-performance liquid chromatography (HPLC) analysis of the culture supernatants, respectively (Fig. 3). In the presence of IPTG, the growth curve has a typical sigmoidal shape with a growth phase of ~ 16 h and a stationary phase beginning when 95% of the medium glycerol has been consumed (Fig. 3A). The growth of *E. coli* cells in the presence of lactose is superimposable with that observed with IPTG, suggesting a similar effect of the two inducers (Fig. 3B). When CWP is used as an inducer, the final optical density is 1.3 higher than that measured with IPTG and lactose (Fig. 3C). This greater biomass could be sustained by the increased availability of nutrients. In this respect, it can be observed that in CWP cultures glycerol is not completely consumed even after 48 h of induction. In addition to the consumption of carbon sources, we looked at the consumption of amino acids, which are both a source of carbon and nitrogen. After 24 h of induction, the three cultures show no differences in amino acid consumption, while after 48 h CWP-induced cells present slightly higher consumption (Fig. 4A).

With regard to GFP production, in the presence of all three inducers, the GFP fluorescence increased over time and the highest signal was observed after 48-h induction (Fig. 3A–C). Overall, lactose and CWP proved to be efficient inducers, quite comparable to IPTG, routinely used on BL21-pET expression systems (Fig. 3). More in detail, lactose induced slightly higher production (specific fluorescence: 89.52 ± 3.23 a.u./mg) compared to IPTG and CWP at the same time (specific fluorescence: 85.81 ± 3.4 a.u./mg and 83.68 ± 2.4 a.u./mg, respectively) (Fig. 3A–C and Additional file 1: Figure S1).

To better compare the three inducers, single-cell distribution of fluorescence was quantified across the bacterial population by flow cytometry. Similar distributions were observed both in terms of cell size and intensity of GFP fluorescence for the three inducers (Additional file 1: Figure S2), with a slight overexpression in the case of lactose (Fig. 3 and Additional file 1: Figure S2). It is well known that highly expressed recombinant proteins in *E. coli* cells often result in the formation of inclusion bodies (IBs) [37, 38]. Therefore, we investigated the formation of IBs of GFP by SDS-PAGE analysis of soluble and insoluble protein fractions extracted after 48-h induction. As shown in Fig. 4B, under all conditions tested, GFP is produced mainly as a soluble protein, indicating that the three inducers behave identically with respect to GFP solubility.

Taken together, these data indicate that CWP acts as an inducer of the BL21-pET system, resulting in a productivity of the model protein GFP that is rather comparable to that obtained with IPTG, which is the inducer of choice for this expression system. To highlight possible physiological differences related to the use of the three inducers, we compared the three cultures in relation to their viability and the expression of stress-related markers.

### CWP alleviates cellular stress associated to GFP overexpression

To study the viability of cells treated with the three inducers, we applied flow cytometry on *E. coli* cell suspensions taken from cultures at the beginning of induction and after 24 and 48 h. Our analyses indicate that the viability of cells treated with IPTG and lactose is ~ 80%, both after 24 and 48 h of induction. In the presence of CWP, cells are even more viable, reaching viability of 92.41 ± 2.29% and 89.57 ± 3.56%, after 24 and 48 h of induction, respectively, (Fig. 4C). These results are in agreement with the achievement of higher biomass in CW-induced cultures and compatible with the hypothesis that the CWP antioxidant properties make cells less susceptible to stress triggered during induction.

It is known that the production of recombinant proteins generates a multitude of stresses, including oxidative stress, which involves the production of reactive oxygen species (ROS), such as superoxide ion and hydrogen peroxide [39, 40]. We studied the effects of the three inducers on oxidative stress by measuring superoxide dismutase (SOD) activity on the soluble fraction of cell lysates. Indeed, the expression of SOD enzymes, and consequently their activity, correlates with the level of intracellular ROS [41, 42]. In the first 4 h of induction, the specific activity of SOD in all cultures increased to ~ 25% of the basal level, thus reaching the highest stress level observed in our experiment (Fig. 4D). At longer induction times (24 and 48 h), different behavior was observed: in IPTG- and lactose-induced cultures, specific activity of SOD returned to the basal level at 24 h and then increased again at 48 h, probably due to a combination of stresses related to recombinant production and aging. On the other hand, the specific activity of SOD in CWP-induced cultures remained below the basal level at both 24 and 48 h, suggesting that the AOC of CWP alleviates oxidative stress.

To study the cellular response during the GFP production, we applied Fourier transform infrared microspectroscopy (micro-FTIR) to intact cells. Micro-FTIR is a powerful approach to investigate in intact cells the modifications in the proteins and lipid components induced by the expression of recombinant proteins [37, 43].

**Fig. 5:**
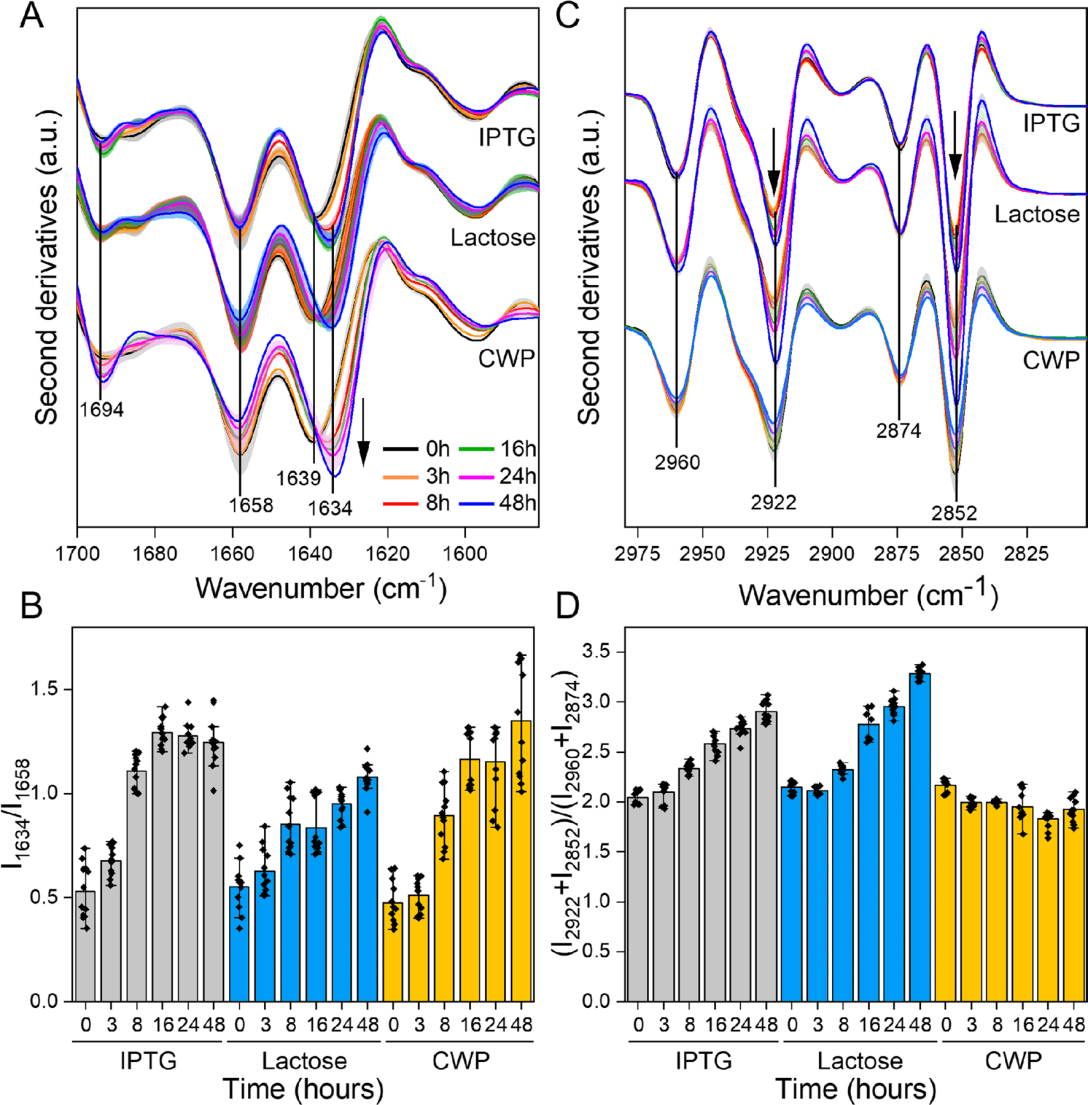
Micro-FTIR analysis of GFP-producing cells. **A** Micro-FTIR analysis of protein secondary structure modifications investigated in the 1700–1580 cm^−1^ spectral range. Second derivative spectra were obtained by averaging at least 4 spectra for each condition. **B** Intensity ratio between the peak of β-sheet (1634 cm^−1^) and that of α-helix (1658 cm^−1^) secondary structures. **C** Micro-FTIR analysis of lipids modifications investigated in the 3000–2805 cm^−1^. Second derivative spectra were obtained by averaging at least 4 spectra for each condition. **D** Intensity ratio between the peaks of CH_2_ stretching (2922 + 2852 cm^−1^) and those of CH_3_ stretching (2960 + 2874 cm^−1^). All experiments were performed in triplicate and the shadowed area (**A**, **C**) and error bars (**B**, **D**) refer to the standard deviation of the data (*n* = 12)

The effects of the three inducers on the whole-cell *E. coli* proteins can be monitored by observing changes in the Amide I band (1700–1580 cm^−1^), mainly due to C=O stretching of the peptide bond. In the absence of any inducer (time 0, in Fig. 5A), the FTIR spectrum displays two main bands, at ~ 1658 cm^−1^, assigned to α-helices and/or random coils, and at ~ 1639 cm^−1^, mainly due to native β-sheets. After 8 h of induction with the three different inducers, we observed a downshift of the band at ~ 1639 cm^−1^ to ~ 1634 cm^−1^, accompanied by an increase in the intensity of the band at ~ 1694 cm^−1^. These spectral features, typical of GFP [44], indicate that the three different inducers cause GFP overexpression. To study its production kinetics, we therefore followed the ratio of the band at 1634 cm^−1^ (β-sheet) to that at ~ 1658 cm^−1^ (α-helix/random coil) as a function of induction times (Fig. 5B). Overall, the micro-FTIR results confirm results previously described (Fig. 3) indicating that IPTG, lactose and CWP are equally effective inducers of recombinant GFP expression.

The effects of the three inducers on cell membrane lipids were investigated by extending micro-FTIR analysis to the spectral range 3050–2800 cm^−1^, mainly due to the absorption of –CH_2_ and –CH_3_ groups of the lipid hydrocarbon chains [45]. In the absence of inducer (time 0 in Fig. 5C), the FTIR spectrum is characterized by four main bands: ~ 2960 cm^−1^ (–CH_3_ asymmetric stretching), ~ 2922 cm^−1^ (–CH_2_ antisymmetric stretching), ~ 2874 cm^−1^ (–CH_3_ symmetric stretching), and ~ 2852 cm^−1^ (–CH_2_ symmetric stretching) [45]. Following induction with IPTG and lactose, an increase in the intensity of the -CH_2_ bands at ~ 2922 cm^−1^ and ~ 2852 cm^−1^ is observed, indicating a significant change in the physico-chemical properties of cellular lipids. In addition, the accumulation of GFP within the cells (Fig. 5B) is accompanied by changes in the ratio of the intensities of the –CH_2_ and –CH_3_ peaks (Fig. 5D), also indicative of lipid membrane perturbations. Overall, these results indicate that in the presence of lactose and IPTG, longer lipid hydrocarbon chains characterize GFP-producing cells, suggesting a change in membrane fluidity and permeability [28, 43]. Remarkably, in the presence of CWP, no significant change in the intensity of the –CH_2_ and –CH_3_ peaks was detected (Fig. 5C and D), indicating that in this case the cell membranes are not significantly altered by GFP production.

Obtained by several orthogonal techniques, our data on recombinant GFP production indicate overall that CWP supports yields comparable to those obtained with IPTG and lactose. Furthermore, the micronutrients of CWP and its AOC appear to positively influence the fitness of GFP-producing cells. As perturbation of the physiological state is one of the main bottlenecks in the production of recombinant proteins, CWP can be considered a generally applicable inducer, however, being particularly suitable for the production of toxic proteins [46].

### CWP alleviates cellular stress associated with recombinant expression of ATX3-Q55

**Fig. 6:**
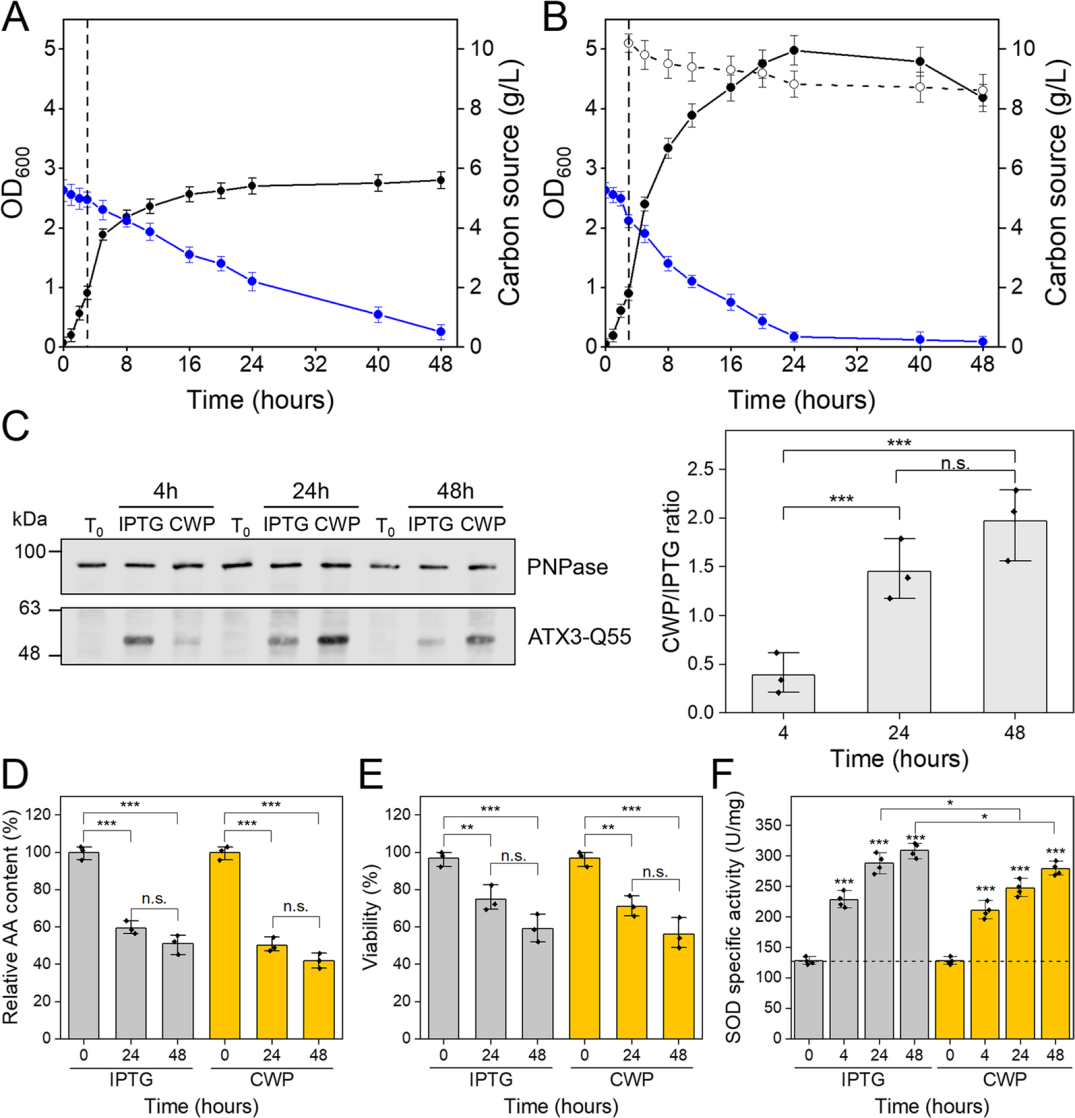
Effects of IPTG and CWP on ATX3-Q55 production and cell physiology. Growth curves of cells overexpressing ATX3-Q55 upon induction with IPTG (**A**) and CWP (**B**). The cell density (full dots, black line, OD_600_), and the concentration of glycerol (blue line) and lactose (empty dots, dotted black line) are plotted in function of growth times. The vertical dotted line indicates the addition of the inducer, error bars indicate the standard deviation of three independent measurements. **C** The production of ATX3-Q55 was monitored by WB analysis on crude extracts from samples taken at the indicated times. One of three independent WB images was shown. The effects of inducers are shown as the ratio of the amount of ATX3-Q55 produced with CWP to that produced with IPTG. The amount of ATX3-Q55 was estimated by densitometric analysis and normalized using PNPase signals. **E** Amino acid consumption determined by HPLC analysis of the culture supernatant from samples taken at the indicated times. **F** Cell viability determined by flow cytometry analysis after labeling of the whole cells with propidium iodine. **F** SOD specific activity measured by McCord and Fridovich assays on the soluble protein fraction extracted from samples taken at the indicated times. Mean values of three (**D**–**F**) or four (**G**) independent measurements are represented with error bars indicating standard deviations. Statistical analyses were performed using unpaired two-tailed Student’s *t*-test, n.s.: not significant *p* > 0.05, *p < 0.05, **p < 0.01, ***p < 0.001

**Fig. 7:**
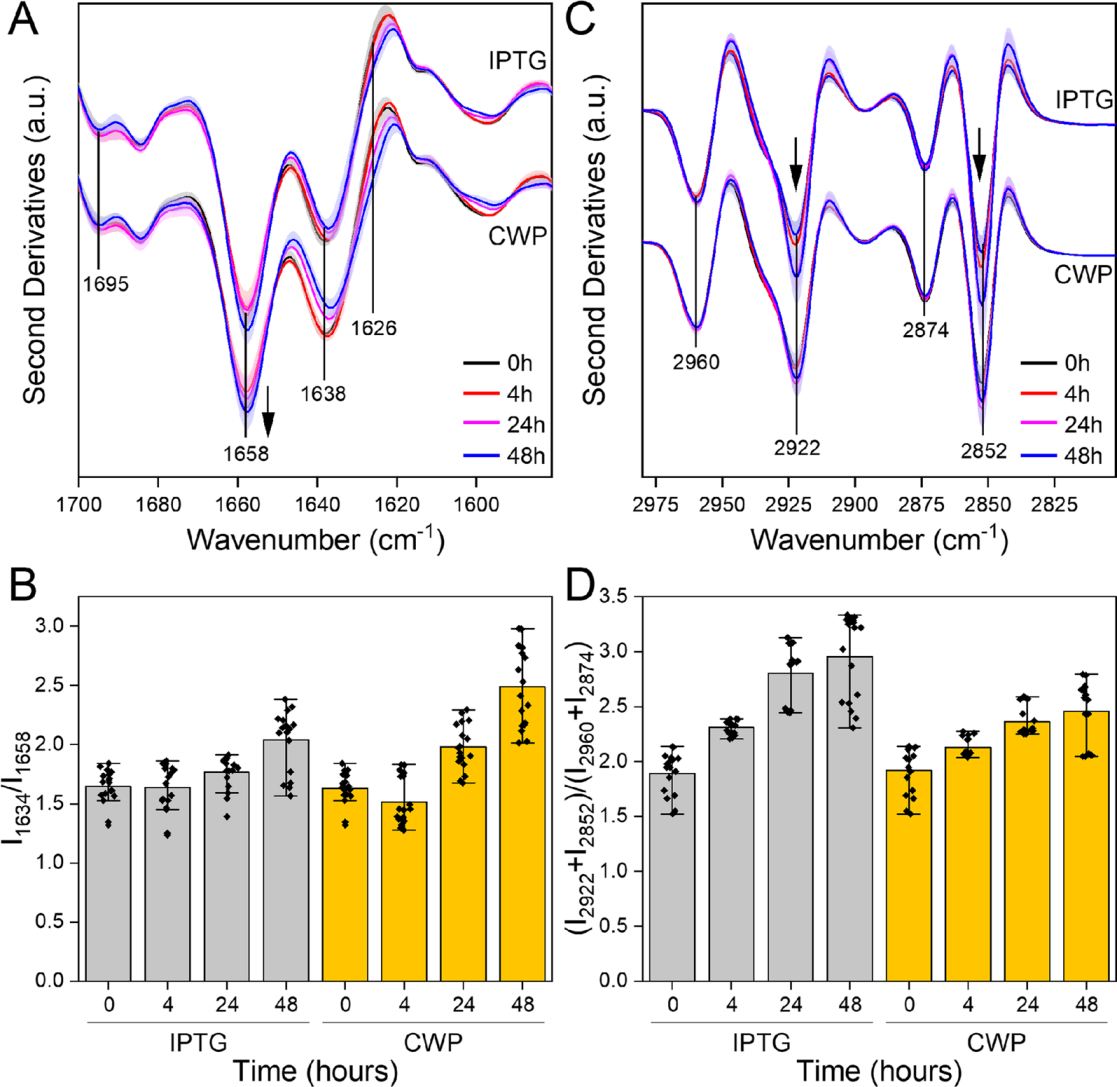
Micro-FTIR analysis of ATX3-Q55-producing cells. **A** Micro-FTIR analysis of protein secondary structure modifications investigated in the 1700–1580 cm^−1^ spectral range. Second derivative spectra were obtained by averaging at least 4 spectra for each condition. **B** Intensity ratio between the peak of β-sheet (1634 cm^−1^) and that of α-helix (1658 cm^−1^) secondary structures. **C** Micro-FTIR analysis of lipids modifications investigated in the 3000–2805 cm^−1^. Second derivative spectra were obtained by averaging at least 4 spectra for each condition. **D** Intensity ratio between the peaks of CH_2_ stretching (2922 + 2852 cm^−1^) and those of CH_3_ stretching (2960 + 2874 cm^−1^). All experiments were performed in triplicate and the shadowed area (**A**, **C**) and error bars (**B**, **D**) refer to the standard deviation of the data (*n* = 12)

To further study the effects of CWP on recombinant protein production, we used CWP as an inducer of the expression of ATX3-Q55, a toxic protein that causes stress and cell damage when expressed in *E. coli* cells [28, 29]. The microbial host and production conditions are the same as those previously described for producing recombinant GFP. In these experiments, we induced the heterologous expression of ATX3-Q55 with IPTG or CWP. The growth curves obtained in the presence of these two inducers show some differences. After the addition of IPTG, the growth phase continued for a further 8 h, reaching a maximum OD_600_ value of 2.6 after 48 h of induction (Fig. 6A). In the presence of CWP, the maximum OD_600_ reached was 4.8 after 24 h of induction (Fig. 6B), slightly decreased to 4.2 after 48 h, probably due to the stresses induced by the expression of ATX3-Q55. Nutrient consumption also shows two different situations between the two inducers: in the presence of IPTG, glycerol is consumed slowly during the kinetics and amino acids are utilized in the first 24 h (blue line in Fig. 6A). With CWP, on the other hand, glycerol is completely utilized in the first 24 h of induction and amino acid consumption is higher than with IPTG. Interestingly, only a small amount of lactose was used during the CWP induction (Fig. 6B). The production of ATX3-Q55 was evaluated by Western blot (WB) analysis on crude extracts and by micro-FTIR on intact cells. WB analysis shows that after 4 h of induction the amount of recombinant protein is slightly higher with IPTG than with CWP. Otherwise, after 24 and 48 h of induction, the amount of ATX3-Q55 is 1.5 and 2 times higher in the presence of CWP than with IPTG (Fig. 6C). Similar results were obtained by analyzing the mean Amide I second derivative spectra of intact cells producing ATX3-Q55 (Fig. 7A and B). Compared to non-induced cells, in the presence of both inducers, the FTIR spectra show an increase in the α-helix/random coil band at ~ 1658 cm^−1^ and a decrease in the native β-sheet band at ~ 1638 cm^−1^ (Fig. 7A). These spectral properties, typical of ATX3-Q55 [28], indicate that ATX3-Q55 was produced recombinantly in slightly higher quantities in the presence of CWP than IPTG (Fig. 7B). For both inducers, long induction times trigger the aggregation of ATX3-Q55, as suggested by the shoulder at ~ 1626 cm^−1^, mainly due to the intermolecular β-sheets typical of protein aggregates (Fig. 7A).

The effects of IPTG and CWP on the physiology of ATX3-Q55-producing cells were investigated as previously described for GFP. Heterologous expression of ATX3-Q55 induces a dramatic reduction in cell viability regardless of the inducer (Fig. 6E). At higher induction times, the specific activity of SOD is lower in CWP-treated cells than in IPTG-treated cells (Fig. 6F). Noteworthy, in the presence of CWP the amount of ATX3-Q55 produced is almost double that of IPTG-treated cells. This higher production of ATX3-Q55 could be due to the AOC of CWP and its ability to alleviate oxidative stress, as indicated by the lower levels of ROS observed in the presence of CWP (Additional file 1: Figure S3). This interpretation is supported by the micro-FTIR analysis in the absorption spectral region of the lipid hydrocarbon chains. As already indicated, the increase in the ratio between the intensities of the CH_2_ and CH_3_ peaks is ascribable to a significant perturbation of the physico-chemical properties of cellular lipids [28, 43]. In cells induced with IPTG, this ratio becomes much more unbalanced than observed in cells induced with CWP (Fig. 7C and D). Overall, even in cultures expressing ATX3-Q55, CWP proves to be a better inducer than IPTG, both in terms of biomass and recombinant protein yield. These effects are presumably related to the lower stress conditions caused by the CWP micronutrients and their antioxidant properties, and the resulting significant prolongation of the post-induction growth phase.

## Discussion

The use of waste materials in new production processes is the essence of the bioeconomy and the zero-waste economy [2, 4]. The problem of CW disposal has long been known, and the extraction of proteins is to date the best way to valorize CW [11, 12]. This process generates a highly polluting secondary waste, i.e., lactose-rich CWP [11, 12]. The biotechnological exploitation of CWP lactose to obtain high value-added compounds (e.g., ethanol, polyhydroxyalkanoate, organic acids, bacteriocin) has been shown to be feasible through enzymatic or in-vivo transformations carried out by microbial cell factories [12, 14, 15, 47–49]. However, only a few natural microorganisms are able to utilize lactose as a carbon source, representing the main bottleneck of CWP valorization [14, 50]. One possible way to valorize CWP is to use it as an inducer of *lac* promoter, as an alternative to lactose and its analogs traditionally used in the production of recombinant proteins in *E. coli* cells. This exploitation of CW and CWP is documented in few works, which mainly focus on comparing the yields of recombinant proteins produced with CWP and traditional inducers such as IPTG or pure lactose [16–19].

In this work, we studied the effects of CWP on the physiology of *E. coli* cells used as hosts for the expression of recombinant proteins such as GFP and ATX3-Q55. In fact, in addition to analyzing the production yield of the two proteins, we evaluated the oxidative stress and cell damage, which are known to be associated with recombinant protein overexpression [39, 46]. The work was carried out comparatively to similar cell cultures exposed to classical inducers such as IPTG and pure lactose. CWP supports good induction levels of both GFP and ATX3-Q55, similar to those obtained with IPTG and lactose. Moreover, CWP improves cell fitness and growth, resulting in longer production periods, decreased membrane stress, increased cell density and viability. These beneficial effects appear to play a key role mainly in the expression of high toxic proteins such as ATX3-Q55. The improvement of cell fitness observed in the presence of CWP is probably due to its micronutrients and high AOC mainly attributable to vitamins (B_2_, B_4_, B_5_ and B_13_), cofactors (flavin adenine dinucleotide) and osmolytes (betaine and choline). It has been documented that adding osmolytes to the culture medium counteracts the formation of inclusion bodies during the recombinant production of proteins [51]. A closer look at the composition of CWP reveals the presence, at low concentration, of organic acids and amino acids, such as citrate, succinate, fumarate and alanine, which may contribute to supporting the growth of *E. coli* BL21 cells [52–54]. The specific role of these compounds identified in CWP will require future systematic and in-depth analysis.

In conclusion, CWP proves to be both a good inducer, promoting production levels comparable to those of traditional inducers, and an excellent supplement to the growth medium, capable of promoting cell fitness challenged by the overexpression of recombinant proteins. Improved cellular fitness enhances the process productivity compared to that observed with classical inducers. Similar results could probably be obtained with the use of growth media supplements, which may come from complex synthesis or purification processes and have considerable market costs. Both of these factors suggest that the use of CWP can reduce the costs of recombinant protein production, especially if the target protein manifests some degree of toxicity towards the expression host. The use of CWP as a low-cost inducer paves the way for the development of new, sustainable, and integrated processes, which starting from the production of extremely fine foods, such as Parmigiano Reggiano or Grana Padano, can lead to the production of industrial proteins and enzymes, without wasting by-products and avoiding additional costs associated with intermediate treatments, component separation or disposal costs.

## Conclusions

This work shows a way for the up-cycling of an agro-food by-product, allocating it to a high value-added process, without applying any expensive or time-consuming pre-treatment. CWP is a cheap inducer of the BL21-pET system, with yields of recombinant protein production comparable to those obtained with traditional inducers. In addition, the CWP micronutrients together with its antioxidant properties play a key role in the improvement of cellular fitness during the stressful heterologous expression, resulting in a greater biomass production.

In conclusion, this work provides a good example of how biotechnology can contribute to closing the circle of a zero-waste economy, not at all conflicting with and harmonizing with long-established industrial processes.

## Materials and methods

### Strains and materials

*E. coli* BL21 (DE3) (EMD Millipore, Billerica, USA) was used as host for recombinant protein production. Plasmids pET21a[GFP] and pET21a[ATX3-Q55] were previously described in [28, 55]. Lactose, tryptone and yeast extract were purchased from Merck (Merck Darmstadt, Germany); IPTG and glycerol were purchased from Genespin (Milano, Italy) and Carlo Erba (Milano, Italy), respectively. CWP was kindly provided by Serum Italia (Cazzago San Martino, Brescia, Italy).

### Characterization of CWP

#### NMR spectroscopy

CWP sample was added of 10% of deuterated phosphate buffer (d-PB, 100 mM pH 7.4) containing 3-(Trimethylsilyl) propionic-2,2,3,3-d_4_ acid sodium salt (d_4_-TSP, final concentration 1 mM) as an internal reference for concentrations and chemical shift. The pH of sample was verified with a microelectrode (Mettler Toledo, Columbus, OH, USA) and adjusted to 7.4 with NaOD or DCl. All pH values were corrected for the isotope effect. All spectra were acquired on an Avance III 600 MHz NMR spectrometer (Bruker, Billerica, MA, USA) equipped with a QCI (^1^H, ^13^C, ^15^N/^31^P and ^2^H lock) cryogenic probe. ^1^H NMR spectra were recorded with *noesygppr1d*, *cpmgpr1d*, *ledbpgppr2s1d* pulse sequences from Bruker library and 256 scans, spectral width 20 ppm, relaxation delay 10 s. The acquisition temperature was 300 K. They were processed with 0.3 Hz line broadening, automatically phased and baseline corrected. Chemical shifts were internally referenced to the TSP peak at 0.0 ppm. The ^1^H,^1^H-TOCSY (TOtal Correlation SpectroscopY) spectra were acquired with 48 scans and 512 increments, a mixing time of 80 ms and a relaxation delay of 2 s. ^1^H, ^13^C-HSQC (Heteronuclear Single Quantum Coherence) spectra were acquired with 128 scans and 512 increments, with a relaxation delay 2 s. NMR spectra processing and peak peaking were done using the MNova software package of Mestrelab (MestReNova v 14.3.0-30573, 2022, Mestrelab Research, Santiago de Compostela, Spain). Metabolites’ identification and peaks’ assignment were done with the support of 2D NMR experiments and comparison with internal and on-line databases (HMDB https://hmdb.ca/, Biological Magnetic Resonance Data Bank https://bmrb.io/, Birmingham Metabolite Library http://www.bml-nmr.org/). The Simple Mixture Analysis (MNova SMA v. 3.0.0.8253) plugin integrated in MNova software was used to set a semi-automatic protocol for the identification and quantification of metabolites. A specific library for the CWP matrix was built, manually refined, and made available as .exp files [33]. The MNova GSD (Global Spectrum Deconvolution) algorithm was employed to deconvolute the overlapping regions, allowing the absolute quantification for metabolites with resonances in crowded spectral areas. Final metabolite concentration was corrected for the dilution factor (10%) and expressed as g/L in CWP.

#### UPLC–HRMS analysis

The UPLC–HRMS analysis was carried by a Waters^®^ Acquity™ UPLC system consisting of a quaternary solvent manager and a sample manager coupled with in-line Waters^®^ photodiode array (PDA) detector, Waters^®^ Xevo G2-XS quadrupole time-of-flight mass spectrometer (MS) and an analytical workstation with Waters^®^ MassLynx™ 4.2 software (Waters, Milford, MA, USA). A 2-μL sample of CWP was injected and separation was carried out with a Waters^®^ Acquity™ Premier HSS T3 column (100 × 2.1 mm I.D., 1.8 μm) coupled with VanGuard™ HSS T3 guard column. The mobile phase consisted of water (A) and acetonitrile (B) both modified with 0.1% formic acid. The eluting condition was isocratic 5% B (0–1 min), linear gradient from 5 to 50% B (1–11 min), 50% to 90% B (11–12 min), then the column was washed with 90% B (12–15 min), and then back to 5% B in (15–16 min) and equilibrate at 5% B for 4 min (16–20 min) before next run. The flow rate was 0.4 mL/min, and the column temperature was maintained at 40 ℃. Mass detection was performed with electrospray ionization (ESI) source operating in positive (ES+) and negative (ES−) ion mode and in sensitivity mode. The capillary voltage and the cone voltage were set to +3/−2 kV and 40 V, respectively. The source temperature was set to 120 °C and the desolvation gas flow was set to 1000 L/h at a temperature of 350 °C, with the cone gas set to 50 L/h. Mass calibration was performed with a sodium formate solution (5 mM), and the peptide leucine enkephalin (*m*/*z* 556.2771 and 554.2615 in ES+ and ES− ion mode, respectively) was used to prepare the lock mass solution. Lock mass solution was diluted to 100 pg/µL in 50% (v/v) aqueous acetonitrile, 0.1% (v/v) formic acid, and injected through a LockSpray interface at 10 µL/min every 30 s, with a reference capillary voltage of +3/−2 kV.

Data were acquired by Full MS scan and data-dependent tandem MS analysis of the five most intense ions (Top 5) over the mass range of 50–1200 m/z (FastDDA experiment). The Full scan survey was applied to trigger MS/MS acquisition of precursor ions with a threshold higher than 5000 intensity per second and the switch back to full scan survey after 5 MS/MS scan. Full scan spectra were acquired with a scan time of 0.2 s and MS/MS spectra acquisition with a scan time of 0.1 s. The dynamic collision energy was set to 6–9 V for 50 Da and 60–80 V for 1200 Da.

The Full MS scan data were processed using MestreNova 14.3.0 (Mnova MS plug-in, Mestrelab Research, Santiago de Compostela, Spain). The raw DDA data were processed with MS-Dial 4.80 (http://prime.psc.riken.jp/compms/msdial/main.html) for ion deconvolution, peak alignment and metabolite identification [56]. The retention time tolerance was set at 0.01 min, and the MS1 and MS2 tolerances, were set at 0.02 Da, and 0.05 Da, respectively. The minimum peak height and the MS/MS abundance threshold were set at 1000 and 10 amplitudes, respectively. The sigma window value was 0.5, and the mass slice width was 0.1 Da. The accurate mass tolerance for identification was 0.005 Da for MS and 0.005 Da for MS/MS. Identification of compounds was based on their calculated accurate mass, isotopic pattern, and structures were confirmed by comparison of MS/MS spectra with the MS-DIAL metabolomics MSP spectral kit (http://prime.psc.riken.jp/compms/msdial/main.html#MSP) and public databases (HMDB https://hmdb.ca/, GNPS https://gnps.ucsd.edu/ and MassBank https://massbank.eu/MassBank/).

#### Evaluation of antioxidant capacity (AOC)

The AOC of the CWP was evaluated as a mean of the total reducing power and radical scavenging ability and measured by Folin-Ciocalteu, 2,2'-azino-bis(3-ethylbenzothiazoline-6-sulfonic acid (ABTS-TEAC) and 2,2-diphenyl-1-picryl-hydrazyl-hydrate (DPPH) spectrophotometric assays as detailed below. Absorbance was measured with a Varian Cary^®^ 50 Scan UV–Visible Spectrophotometer (Agilent, Santa Clara, CA, USA) and related to a blank solution. Data were reported as means (± SD) of triplicate measurements in two independent evaluations (*n* = 6); a lactose solution (170 g/L in water) was used as a reference.

The total reducing power was determined with the Folin Ciocalteu assay using 8 μL of CWP sample or lactose solution as described in [31]. Standard solutions (0–200 μg/mL) of gallic acid were used for calibration (linear fitting *R*^2^ > 0.99, *n* = 7). Results were expressed as mg of gallic acid equivalent (GAE eq) per liter of CWP.

The radical scavenging ability was determined by ABTS-TEAC and DPPH assay. The ABTS-TEAC assay is based on the evaluation of the scavenging capacity of an antioxidant to the long-life colored cation ABTS^+^, while the DPPH assay is based on the scavenging of the stable free-radical 2,2-diphenyl-1-picrylhydrazyl. Analyses were carried out as described in [31] using 15- and 50-μL samples of CWP for the ABTS and DPPH assay, respectively. Standard solutions (0–500 μM) of Trolox (6-hydroxy-2,5,7,8-tetramethylchroman-2-carboxylic acid, a water-soluble analogue of vitamin E) were used for calibration (linear fitting *R*^2^ > 0.99, *n* = 7). Results of both radical scavenging assays were expressed as µmol of Trolox equivalent (TE) per liter of CWP.

### Culture conditions for recombinant production of GFP and ATX3-Q55 protein in *E. coli*

The plasmid constructs (pET21 [GFP] and pET21 [ATX3-Q55]) were used to transform *E. coli* BL21(DE3). Recombinant proteins were produced in Lennox medium (LM, yeast extract 5 g/L, tryptone 10 g/L, NaCl 5 g/L) modified with the addition of glycerol 5 g/L (LM-G) and supplemented with ampicillin (100 mg/L). Pre-cultures were grown in LM-G to OD_600_ ~ 0.6–0.8 and diluted 1:25 in 100 mL of fresh LM-G in 500-mL flasks. Cultures were grown in LM-G at 37 °C to OD_600_ 0.8–1.0 and induced with 0.1 g/L IPTG, or 10 g/L lactose or CWP (diluted to the lactose final concentration of 10 g/L), at 25 °C for 48 h (Fig. 1).

### Sample collection and preparation

Time series experiments were carried out to profile each culture in terms of cell growth, production of recombinant proteins, nutrient consumption, and cell viability. More in detail, cell growth was monitored by measuring OD_600_ with a Jasco V-730 UV/NIR spectrophotometer (Jasco Europe, Cremella, Lecco, Italy). For each culture, at each selected time point, a volume of cell culture corresponding to 20 OD_600_ was collected by centrifugation at 1000*g* for 10 min at 4 °C. The supernatant was analyzed by HPLC to determine metabolites and amino acid compositions, as detailed below.

Cell pellets were washed three times and gently resuspended in 1 mL of cold physiological solution (0.9% NaCl w/v in Milli-Q water). Whole-cell analyses were carried out on cell suspensions, while crude extracts were prepared by mechanically lysing the cells with glass beads. Glass beads were added to 1 mL of cell suspension and the samples were subjected to 5 cycles of 1 min vortex and 1 min ice. Then, the crude extract was centrifuged at 3000*g* for 10 min at 4 °C to obtain the soluble and insoluble protein fractions. The total protein content (*P*_tot_) of the soluble protein fraction was determined by the Bradford protein assay (Bio-Rad, Hercules, CA, USA), using bovine serum albumin as the standard. The soluble and insoluble protein fractions were also used to assay the recombinant protein production, as detailed below.

### Monitoring the production of GFP

GFP production was assessed on the soluble protein fraction by monitoring fluorescence emission at 528 nm with a Cary Eclipse (Varian Inc., Palo Alto, CA, USA) spectrofluorometer using an excitation wavelength of 475 nm. The specific fluorescence was calculated using the following equation:

$${\text{Specific\,fluorescence}} \, ({\text{a.u./mg}}) = \frac{{{\text{Fluorescence\,at}}\;528\;{\text{nm}}\;({\text{a.u.}})}}{{P_{tot} \;({\text{mg}})}}10^{ - 3}$$

GFP production was also tested on whole cells by flow cytometry with a CytoFlex S cytofluorimeter equipped with argon ion laser at 488 nm (Beckman Coulter, Life Sciences, Indianapolis, IN, USA). Analyses were carried out on cell suspension acquiring 10,000 events.

At the end of the induction phase (48 h), GFP production was analyzed by SDS-PAGE. The crude extracts, soluble and insoluble protein fractions were denatured by adding Laemmli buffer (60 mM Tris-Cl pH 6.8, 2% w/v SDS, 10% v/v glycerol, 5% v/v β-mercaptoethanol, 0.02‰ w/v bromophenol blue) and boiling the samples at 99 °C for 5 min. Balanced amounts of these samples, corresponding to a OD_600_ ~ 0.15, were loaded on 14% SDS-PAGE and proteins visualized after staining with Gel-Code Blue (ThermoFisher, Waltham, MA, USA).

### Monitoring the production of ATX3-Q55

The production of ATX3-Q55 was monitored by WB analysis. Crude extracts were obtained from aliquots of OD_600_ ~ 4 of cells collected at different growth times according to the procedure previously described [28] and denatured in the Laemmli buffer as previously described. A preliminary SDS-PAGE analysis was carried out to determine the amount of crude extracts used for WB analysis. Comparable amount of crude extracts (~ OD_600_: 0.20) was loaded on 12% SDS-PAGE and then blotted on Odyssey nitrocellulose membrane (LI-COR Biosciences, Lincoln, NE, USA) using the Mini Protean electroblotting system (Bio-Rad, Hercules, CA, USA) and Towbin transfer buffer (25 mM Tris, 192 mM glycine, 20% (v/v) methanol, pH 8.3) (constant current 400 mA for 2 h at 4 °C). Rabbit polyclonal primary anti-AT3 Z46 (1:5000) and anti-PNPase (1:150,000) antibodies were diluted in 5% milk and incubated overnight; anti-rabbit fluorescent IRDye^®^ 800CW (LI-COR Biosciences, Lincoln, NE, USA) was used as a secondary antibody for both primary antibodies. Signal quantification was carried out using Image Studio software (LI-COR Biosciences, Lincoln, NE, USA). The experiment was performed in triplicate.

### Analysis of metabolites and amino acid consumption

The supernatant of cultures expressing GFP was analyzed by HPLC to determine metabolites and amino acid compositions. Concentrations of lactose, glycerol and amino acids were determined with an HPLC system equipped with a Jasco PU-2080 pump, Jasco UV-1575 and Jasco RI-1530 detectors (Jasco Europe, Cremella, Lecco, Italy). The injection volume of samples and standards was 20  μL. Lactose and glycerol were monitored on an Aminex-HPX‐87H column (Bio-Rad Laboratories, Hercules, CA, USA) protected by a Micro-Guard Cation H cartridge (Bio-Rad Laboratories, Hercules, CA, USA). The analyses were performed isocratically at 0.5 mL/min and 55 °C. The mobile phase consisted of 5 mM H_2_SO_4_ and the sugars were detected with a refractive index detector. Amino acid analyses were carried out as described in [57] with some modifications. Amino acids were derivatized with *o*-phthalaldehyde prepared by dissolving 25 mg of *o*-phthalaldehyde and 25 mg of 3-mercaptopropionic acid in 5 mL of 0.2 M borate buffer, pH 10.2. The resulting derivatization reagent was flushed in nitrogen and stored in the dark at 4 °C. The derivatization reaction (200 μL) was started by adding 5.5 μL of derivatization reagent to 5.5 μL of sample. Derivatized amino acids were separated with a XTerra RP18 column (4.6 mm × 250 mm i.d, 5 μm particle size) (Waters, Milford, MA, USA) equipped with a SecurityGuard precolumn (Phenomenex, Torrance, CA, USA). Separation was performed at 40 °C with a mobile phase flow rate of 1 mL/min. HPLC separation was performed with mobile phases A (40 mM sodium phosphate buffer, pH 7.8) and B (30% acetonitrile, 60% methanol and 10% H_2_O). The column was equilibrated with 94.5% (v/v) A: 5.5% (v/v) B for 10 min. The elution gradients were set as follows: 94.5%/5.5% for 0.85 min; 2.15 min linear gradient to 13% phase B; 23 min linear gradient to 54% phase B; 94.5%/5.5% for 4 min. The peaks were identified by comparison with the amino acid retention times of a standard solution of amino acids in 0.1 M HCl.

### Determination of oxidative stress

SOD activity was measured on soluble protein fractions according to the procedure of McCord and Fridovich [58] with a Jasco V-730 UV/NIR spectrophotometer (Jasco Europe, Cremella, Lecco, Italy). One unit of SOD is defined as the amount of protein required to inhibit the reduction of cytochrome *C* by the superoxide radical by approximately 50%. The SOD specific activity was calculated using the following equation:

$${\text{SOD specific activity (U/mg)}} = \frac{{{\text{SOD}}\;{\text{activity}}({\text{U}})}}{{P_{tot} ({\text{mg}})}}$$

Experiments were performed in quadruplicate.

Intracellular ROS levels were determined on ATX3-Q55-producing cells using 2,7-dichlorofluorescein diacetate (DCF-DA; Sigma–Aldrich Co., St. Louis, MO, USA). DCF-DA is a permeable probe that is oxidized into the fluorescent product dichlorofluorescein (DCF) in the presence of ROS. Before and after 24- and 48-h induction with IPTG and CWP, cells were harvested, washed twice and resuspended in saline phosphate buffer. Aliquots of cells (~ 0.2 OD_600_) were incubated with 50 μM DCF-DA for 30 min at 37 °C. Fluorescence values were measured at 538 nm (excitation: 485 nm) with the Viktor 3 microplate reader (PerkinElmer, Waltham, MA, USA). The experiment was carried out in triplicate.

### Micro-FTIR analysis

The effects of different inducers on whole cells were investigated by micro-FTIR as described in [28]. A few microliters (~ 2 μL) of the cell suspension were deposited onto a BaF_2_ window and dried at room temperature for ~ 30 min to remove excess water. FTIR absorption spectra were acquired in transmission mode, in the spectral range 4000–800 cm^−1^, by a Varian 610-IR infrared microscope coupled to a Varian 670-IR FTIR spectrometer (both from Varian Australia Pty Ltd., Mulgrave, VIC, Australia), equipped with a mercury cadmium telluride nitrogen-cooled detector. The variable aperture of the microscope was adjusted to ~ 200 μm × 200 μm and spectra were collected with a spectral resolution of 2.0 cm^−1^, 25 kHz scan speed, triangular apodization and accumulation of 512 scan co-additions.

For comparison, the spectra—corrected for residual water vapor absorption—were normalized to the area of Amide I band and the analysis of the second derivative was performed (after a 13-point smoothing of the measured spectra) by the Savitzky–Golay method (3rd polynomial, 9 smoothing points), using GRAMS/32 software (Galactic Industries Corporation, Salem, NH, USA).

It should be noted that the absolute intensities of the peaks cannot be obtained from the spectra of the second derivatives. However, provided the original absorption spectra are of high quality, the second derivatives can be used to monitor spectral changes if the very same analyses are performed on the original data [59–61].

For each sample, to assess spectral heterogeneity, we repeated several measurements by selecting different areas on the same sample (5–7 spectra for each condition in each experiment), taking advantage of the variable diaphragm aperture of the infrared microscope. Furthermore, to evaluate the reproducibility of the results, we performed three independent experiments.

### Assessment of cell viability

The viability of *E. coli* cells before and after induction for 24 and 48 h was determined by flow cytometry with a CytoFlex cytofluorimeter equipped with a 488 nm Argon ion laser (Beckman Coulter, Life Science, Indianapolis, IN, USA). Dead cells were detected by monitoring the fluorescence of propidium iodine (5 μg/mL). Control samples of dead cells were prepared by incubating the cells for 10 min in 70% EtOH before staining with propidium iodine. Analyses were carried out by acquiring 10,000 events. To analyze the viability of ATX3-Q55-producing cells, a forward scatter threshold of 30,000 was set, due to the small size of these cells.

### Statistical analysis

Unless otherwise indicated, experiments were performed in triplicate. Statistical analysis was performed using OriginLab software (OriginLab Corporation, Northampton, USA). The *p*-values were determined using an unpaired two-tailed *t*-test. No statistical methods or criteria were used to estimate size or to include or exclude samples.

### Supplementary Information

**Additional file 1: Figure S1.** GFP production after 48 h of induction with different inducers. (A) GFP production was monitored by measuring the fluorescence intensity on the crude extracts. (B) GFP production by a single cell was determined by flow cytometry from whole cells. Mean values of three (A) or four (B) independent measurements are represented with error bars indicating standard deviations. Statistical analyses were performed using unpaired two-tailed Student's t-test, only significant changes (*p < 0.05) are reported. **Figure S2.** Flow cytometry analysis of GFP-producing cells. BL21 (DE3) *E. coli* cells overexpressing GFP were analyzed before the induction (time 0) and after 6 h, 24 h and 48 h after the induction with IPTG (A), lactose (B), and CWP (C), at 25 °C. GFP fluorescence was plotted in function of forward scatter (FSC), which is proportional to cell size. One of three independent measurements was shown. **Figure S3.** Effects of IPTG and CWP on ROS generation in cells producing ATX3-Q55. ROS generation was monitored using DCF-DA, a permeable probe that is oxidized in the presence of ROS forming the fluorescent compound DCF. Mean values of three independent measurements are shown with error bars indicating standard deviations. Statistical analyses were performed using unpaired two-tailed Student’s *t*-test, *p < 0.05, **p < 0.01, ***p < 0.001.

## Data Availability

All data generated or analysed during this study are included in this published article and its additional information files.

## Abbreviations

ABTS: 2,2'-azino-bis(3-ethylbenzothiazoline-6-sulfonic acid
AOC: Antioxidant capacity
ATX3-Q55: Ataxin-3 Q55 variant
CW: Cheese whey
CWP: Cheese-whey permeate
DPPH: 2,2-diphenyl-1-picryl-hydrazyl-hydrate
ESI: Electrospray ionization
IB: Inclusion body
IPTG: Isopropyl β-d-thiogalactopyranoside
GFP: Green fluorescent protein
HPLC: High-performance liquid chromatography
LM: Lennox medium
LM-G: Lennox medium with 5 g/L glycerol
Micro-FTIR: Fourier transform infrared microspectroscopy
MS: Mass spectrometry
ROS: Reactive oxygen species
UPLC-HR-MS: Ultra-performance liquid chromatography coupled with high resolution mass spectrometry
SMA: Simple mixture analysis
SOD: Superoxide dismutase
WB: Western blot

## References

[1] Branduardi P (2021). Closing the loop: the power of microbial biotransformations from traditional bioprocesses to biorefineries, and beyond. Microb Biotechnol.

[2] Sharma P, Gaur VK, Sirohi R, Varjani S, Hyoun Kim S, Wong JWC (2021). Sustainable processing of food waste for production of bio-based products for circular bioeconomy. Bioresour Technol.

[3] Sindhu R, Binod P, Nair RB, Varjani S, Pandey A, Gnansounou E. Waste to wealth. In: Current developments in biotechnology and bioengineering. Elsevier; 2020. p. 181–97.

[4] Ravindran R, Jaiswal AK (2016). Exploitation of food industry waste for high-value products. Trends Biotechnol.

[5] van der Wiel BZ, Weijma J, van Middelaar CE, Kleinke M, Buisman CJN, Wichern F (2019). Restoring nutrient circularity: a review of nutrient stock and flow analyses of local agro-food-waste systems. Resour Conserv Recycl X.

[6] Dey D, Richter JK, Ek P, Gu B-J, Ganjyal GM (2021). Utilization of food processing by-products in extrusion processing: a review. Front Sustain Food Syst.

[7] Mateos-Aparicio I, Matias A, Galanakis CM (2019). Food industry processing by-products in foods. The role of alternative and innovative food ingredients and products in consumer wellness.

[8] Souza AFC, Gabardo S, Coelho RdJS (2022). Galactooligosaccharides: physiological benefits, production strategies, and industrial application. J Biotechnol.

[9] Albizzati PF, Tonini D, Astrup TF (2021). High-value products from food waste: an environmental and socio-economic assessment. Sci Total Environ.

[10] Kieliszek M, Piwowarek K, Kot AM, Pobiega K (2020). The aspects of microbial biomass use in the utilization of selected waste from the agro-food industry. Open Life Sci.

[11] Marwaha SS, Kennedy JF (2007). Whey-pollution problem and potential utilization. Int J Food Sci Technol.

[12] Siso MIG (1996). The biotechnological utilization of cheese whey: a review. Bioresour Technol.

[13] Illanes A. Lactose. In: Lactose-derived prebiotics. Elsevier; 2016. p. 1–33.

[14] Amaro TMMM, Rosa D, Comi G, Iacumin L (2019). Prospects for the use of whey for polyhydroxyalkanoate (PHA) production. Front Microbiol.

[15] Donzella S, Fumagalli A, Arioli S, Pellegrino L, D’Incecco P, Molinari F (2022). Recycling food waste and saving water: optimization of the fermentation processes from cheese whey permeate to yeast oil. Fermentation.

[16] De León-Rodríguez A, Rivera-Pastrana D, Medina-Rivero E, Flores-Flores JL, Estrada-Baltazar A, Ordóñez-Acevedo LG (2006). Production of penicillin acylase by a recombinant *Escherichia coli* using cheese whey as substrate and inducer. Biomol Eng.

[17] Hausjell J, Miltner M, Herzig C, Limbeck A, Saracevic Z, Saracevic E (2019). Valorisation of cheese whey as substrate and inducer for recombinant protein production in *E. coli* HMS174(DE3). Bioresour Technol Rep.

[18] Mobayed FH, Nunes JC, Gennari A, de Andrade BC, Ferreira MLV, Pauli P (2021). Effect of by-products from the dairy industry as alternative inducers of recombinant β-galactosidase expression. Biotechnol Lett.

[19] Viitanen MI, Vasala A, Neubauer P, Alatossava T (2003). Cheese whey-induced high-cell-density production of recombinant proteins in Escherichia coli. Microb Cell Factories.

[20] Gasset A, Garcia-Ortega X, Garrigós-Martínez J, Valero F, Montesinos-Seguí JL (2022). Innovative bioprocess strategies combining physiological control and strain engineering of *Pichia pastoris* to improve recombinant protein production. Front Bioeng Biotechnol.

[21] Rosano GL, Ceccarelli EA. Recombinant protein expression in *Escherichia coli*: advances and challenges. Front Microbiol. 2014;5. 10.3389/fmicb.2014.00172 PMC4029002 24860555

[22] Rosano GL, Morales ES, Ceccarelli EA (2019). New tools for recombinant protein production in *Escherichia coli* : a 5-year update. Protein Sci.

[23] Studier FW, Moffatt BA (1986). Use of bacteriophage T7 RNA polymerase to direct selective high-level expression of cloned genes. J Mol Biol.

[24] Mierendorf RC, Morris BB, Hammer B, Novy RE, Reischl U (1997). Expression and purification of recombinant proteins using the pET system. Molecular diagnosis of infectious diseases.

[25] Studier FW (2005). Protein production by auto-induction in high-density shaking cultures. Protein Expr Purif.

[26] Alfasi S, Sevastsyanovich Y, Zaffaroni L, Griffiths L, Hall R, Cole J (2011). Use of GFP fusions for the isolation of *Escherichia coli* strains for improved production of different target recombinant proteins. J Biotechnol.

[27] Sevastsyanovich Y, Alfasi S, Overton T, Hall R, Jones J, Hewitt C (2009). Exploitation of GFP fusion proteins and stress avoidance as a generic strategy for the production of high-quality recombinant proteins. FEMS Microbiol Lett.

[28] Ami D, Sciandrone B, Mereghetti P, Falvo J, Catelani T, Visentin C (2021). Pathological ATX3 expression induces cell perturbations in *E. coli* as revealed by biochemical and biophysical investigations. Int J Mol Sci.

[29] Invernizzi G, Aprile FA, Natalello A, Ghisleni A, Penco A, Relini A (2012). The relationship between aggregation and toxicity of polyglutamine-containing ataxin-3 in the intracellular environment of *Escherichia coli*. PLoS ONE.

[30] Ciaramelli C, Palmioli A, Angotti I, Colombo L, De Luigi A, Sala G (2022). NMR-driven identification of cinnamon bud and bark components with anti-Aβ activity. Front Chem.

[31] Palmioli A, Mazzoni V, De Luigi A, Bruzzone C, Sala G, Colombo L (2022). Alzheimer’s disease prevention through natural compounds: cell-free, in vitro, and in vivo dissection of hop (*Humulus lupulus* L.) multitarget activity. ACS Chem Neurosci.

[32] Palmioli A, Bertuzzi S, De Luigi A, Colombo L, La Ferla B, Salmona M (2019). bioNMR-based identification of natural anti-Aβ compounds in Peucedanum ostruthium. Bioorg Chem.

[33] Palmioli A, Airoldi C. SMA library for metabolites identification and quantification in cheese whey permeate (CWP). 2022. 10.17632/xn5ntwbtjz.1.

[34] Dams RI, Viana MB, Guilherme AA, Silva CM, dos Santos AB, Angenent LT (2018). Production of medium-chain carboxylic acids by anaerobic fermentation of glycerol using a bioaugmented open culture. Biomass Bioenergy.

[35] Martínez-Gómez K, Flores N, Castañeda HM, Martínez-Batallar G, Hernández-Chávez G, Ramírez OT (2012). New insights into *Escherichia coli* metabolism: carbon scavenging, acetate metabolism and carbon recycling responses during growth on glycerol. Microb Cell Factories.

[36] Murarka A, Dharmadi Y, Yazdani SS, Gonzalez R (2008). Fermentative utilization of glycerol by *Escherichia coli* and its implications for the production of fuels and chemicals. Appl Environ Microbiol.

[37] Doglia SM, Ami D, Natalello A, Gatti-Lafranconi P, Lotti M (2008). Fourier transform infrared spectroscopy analysis of the conformational quality of recombinant proteins within inclusion bodies. Biotechnol J.

[38] Slouka C, Kopp J, Hutwimmer S, Strahammer M, Strohmer D, Eitenberger E (2018). Custom made inclusion bodies: impact of classical process parameters and physiological parameters on inclusion body quality attributes. Microb Cell Factories.

[39] Hoffmann F, Rinas U, Enfors SO (2004). Stress induced by recombinant protein production in * Escherichia coli *. Physiological stress responses in bioprocesses.

[40] Striedner G, Cserjan-Puschmann M, Potschacher F, Bayer K (2003). Tuning the transcription rate of recombinant protein in strong *Escherichia coli* expression systems through repressor titration. Biotechnol Prog.

[41] Baez A, Shiloach J (2013). Escherichia coli avoids high dissolved oxygen stress by activation of SoxRS and manganese-superoxide dismutase. Microb Cell Factories.

[42] Imlay JA (2008). Cellular defenses against superoxide and hydrogen peroxide. Annu Rev Biochem.

[43] Ami D, Natalello A, Schultz T, Gatti-Lafranconi P, Lotti M, Doglia SM (2009). Effects of recombinant protein misfolding and aggregation on bacterial membranes. Biochim Biophys Acta BBA - Proteins Proteomics.

[44] Roca-Pinilla R, Fortuna S, Natalello A, Sánchez-Chardi A, Ami D, Arís A (2020). Exploring the use of leucine zippers for the generation of a new class of inclusion bodies for pharma and biotechnological applications. Microb Cell Factories.

[45] Casal HL, Mantsch HH (1984). Polymorphic phase behaviour of phospholipid membranes studied by infrared spectroscopy. Biochim Biophys Acta BBA - Rev Biomembr.

[46] Singha TK, Gulati P, Mohanty A, Khasa YP, Kapoor RK, Kumar S (2017). Efficient genetic approaches for improvement of plasmid based expression of recombinant protein in *Escherichia coli*: a review. Process Biochem.

[47] Boumaiza M, Colarusso A, Parrilli E, Garcia-Fruitós E, Casillo A, Arís A (2018). Getting value from the waste: recombinant production of a sweet protein by *Lactococcus lactis* grown on cheese whey. Microb Cell Factories.

[48] Gabardo S, Rech R, Rosa CA, Ayub MAZ (2014). Dynamics of ethanol production from whey and whey permeate by immobilized strains of *Kluyveromyces marxianus* in batch and continuous bioreactors. Renew Energy.

[49] Gabardo S, Pereira GF, Klein MP, Rech R, Hertz PF, Ayub MAZ (2016). Dynamics of yeast immobilized-cell fluidized-bed bioreactors systems in ethanol fermentation from lactose-hydrolyzed whey and whey permeate. Bioprocess Biosyst Eng.

[50] Koller M, Hesse P, Bona R, Kutschera C, Atlić A, Braunegg G (2007). Potential of various archae- and eubacterial strains as industrial polyhydroxyalkanoate producers from whey. Macromol Biosci.

[51] de Marco A, Vigh L, Diamant S, Goloubinoff P (2005). Native folding of aggregation-prone recombinant proteins in Escherichia coli by osmolytes, plasmid- or benzyl alcohol-overexpressed molecular chaperones. Cell Stress Chaperones.

[52] Blommel PG, Becker KJ, Duvnjak P, Fox BG (2008). Enhanced bacterial protein expression during auto-induction obtained by alteration of lac repressor dosage and medium composition. Biotechnol Prog.

[53] Li Z, Kessler W, van den Heuvel J, Rinas U (2011). Simple defined autoinduction medium for high-level recombinant protein production using T7-based *Escherichia coli* expression systems. Appl Microbiol Biotechnol.

[54] Sivashanmugam A, Murray V, Cui C, Zhang Y, Wang J, Li Q (2009). Practical protocols for production of very high yields of recombinant proteins using *Escherichia coli*. Protein Sci.

[55] Tedeschi G, Mangiagalli M, Chmielewska S, Lotti M, Natalello A, Brocca S (2017). Aggregation properties of a disordered protein are tunable by pH and depend on its net charge per residue. Biochim Biophys Acta BBA - Gen Subj.

[56] Tsugawa H, Cajka T, Kind T, Ma Y, Higgins B, Ikeda K (2015). MS-DIAL: data-independent MS/MS deconvolution for comprehensive metabolome analysis. Nat Methods.

[57] Rutherfurd SM, Gilani GS (2009). Amino acid analysis. Curr Protoc Protein Sci.

[58] McCord JM, Fridovich I (1969). Superoxide dismutase. J Biol Chem.

[59] Ami D, Franco AR, Artusa V, Mereghetti P, Peri F, Natalello A (2022). A global picture of molecular changes associated to LPS treatment in THP-1 derived human macrophages by fourier transform infrared microspectroscopy. Int J Mol Sci.

[60] Ami D, Duse A, Mereghetti P, Cozza F, Ambrosio F, Ponzini E (2021). Tear-based vibrational spectroscopy applied to amyotrophic lateral sclerosis. Anal Chem.

[61] Zhang J, Yan Y-B (2005). Probing conformational changes of proteins by quantitative second-derivative infrared spectroscopy. Anal Biochem.

