# Supplementary material for: Cheese-whey permeate improves the fitness of Escherichia coli cells during recombinant protein production
Source: Biotechnol Biofuels Bioprod. 2023 Feb 23;16:30. doi: 10.1186/s13068-023-02281-8 (PMC9948444; doi:10.1186/s13068-023-02281-8)
Supplement: Supplementary file 1 — Additional file 1: Figure S1. GFP production after 48 h of induction with different inducers. (A) GFP production was monitored by measuring the fluorescence intensity on the crude extracts. (B) GFP production by a single cell was determined by flow cytometry from whole cells. Mean values of three (A) or four (B) independent measurements are represented with error bars indicating standard deviations. Statistical analyses were performed using unpaired two-tailed Student's t-test, only significant changes (*p < 0.05) are reported. Figure S2. Flow cytometry analysis of GFP-producing cells. BL21 (DE3) E. coli cells overexpressing GFP were analyzed before the induction (time 0) and after 6 h, 24 h and 48 h after the induction with IPTG (A), lactose (B), and CWP (C), at 25 °C. GFP fluorescence was plotted in function of forward scatter (FSC), which is proportional to cell size. One of three independent measurements was shown. Figure S3. Effects of IPTG and CWP on ROS generation in cells producing ATX3-Q55. ROS generation was monitored using DCF-DA, a permeable probe that is oxidized in the presence of ROS forming the fluorescent compound DCF. Mean values of three independent measurements are shown with error bars indicating standard deviations. Statistical analyses were performed using unpaired two-tailed Student’s t-test, *p < 0.05, **p < 0.01, ***p < 0.001. [file 13068_2023_2281_MOESM1_ESM.docx]

**Supplementary material**

**Figure S1. GFP production after 48 hours of induction with different inducers. A**) GFP production was monitored by measuring the fluorescence intensity on the crude extracts. **B)** GFP production by a single cell was determined by flow cytometry from whole cells. Mean values of three (A) or four (B) independent measurements are represented with error bars indicating standard deviations. Statistical analyses were performed using unpaired two-tailed Student's t-test, only significant changes (*: p < 0.05) are reported.

**
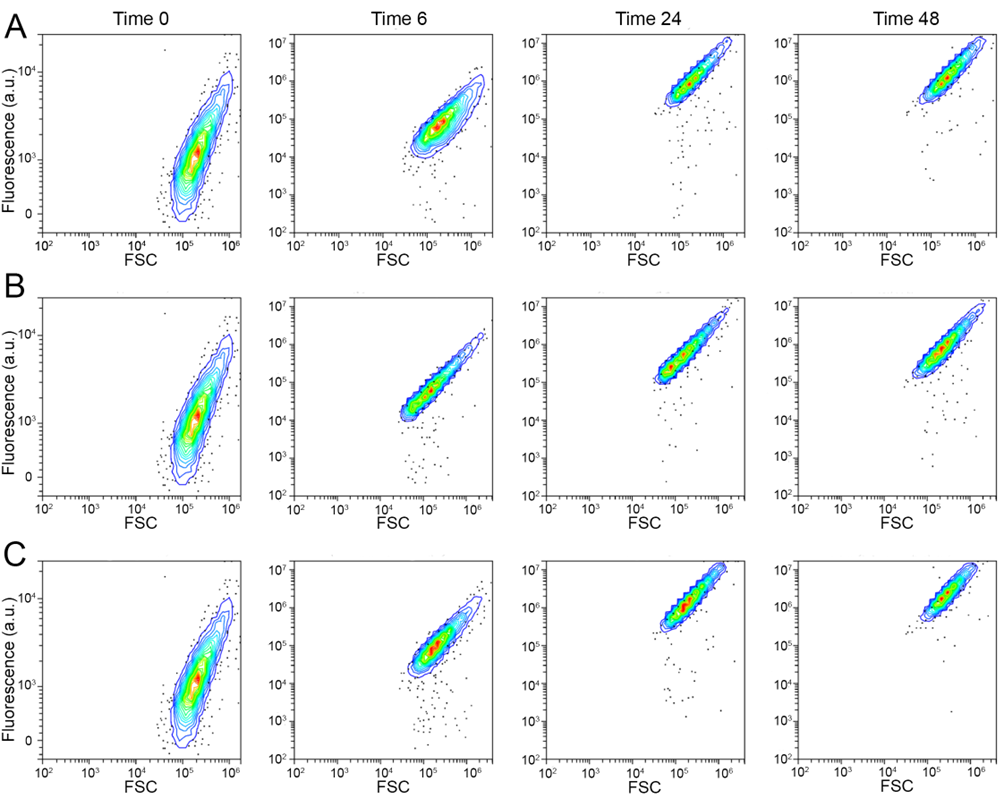
**

**Figure S2.** **Flow cytometry analysis of GFP-producing cells.** BL21 (DE3) *E. coli* cells overexpressing GFP were analyzed before the induction (time 0) and after 6 hours, 24 hours and 48 hours after the induction with IPTG (A), lactose (B), and CWP (C), at 25°C. The GFP fluorescence was plotted in function of forward scatter (FSC), which is proportional to the size of the cell. One of three independent measurements was shown.

**Figure S3. Effects of IPTG and CWP on ROS generation in cells producing ATX3-Q55.** ROS generation was monitored using DCF-DA, a permeable probe that is oxidized in the presence of ROS forming the fluorescent compound DCF. Mean values of three independent measurements are shown with error bars indicating standard deviations. Statistical analyses were performed using unpaired two-tailed Student's *t*-test, **p* < 0.05, ***p* < 0.01, ****p* < 0.001.
